# Trap States in Reduced Colloidal Titanium Dioxide Nanoparticles Have Different Proton Stoichiometries

**DOI:** 10.1021/acscentsci.4c01074

**Published:** 2024-11-22

**Authors:** Noreen E. Gentry, Noah J. Gibson, Justin L. Lee, Jennifer L. Peper, James M. Mayer

**Affiliations:** Department of Chemistry, Yale University, New Haven, Connecticut 06520-8107, United States

## Abstract

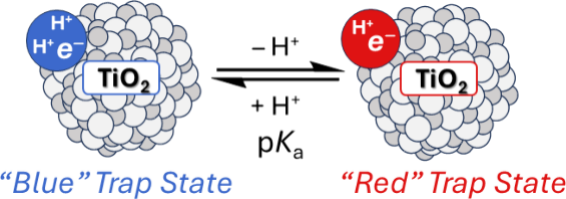

Added electrons and holes in semiconducting (nano)materials typically occupy “trap states,” which often determine their photophysical properties and chemical reactivity. However, trap states are usually ill-defined, with few insights into their stoichiometry or structure. Our laboratory previously reported that aqueous colloidal TiO_2_ nanoparticles prepared from TiCl_4_ + H_2_O have two classes of electron trap states, termed **Blue** and **Red**. Herein, we show that the formation of **Red** from oxidized TiO_2_ requires 1*e*^–^ + 1H^+^, while **Blue** requires 1*e*^–^ + 2H^+^. The two states are in a protic equilibrium, **Blue** ⇌ **Red** + H^+^, with *K*_eq_ = 2.65 mM. The **Blue** states in the TiO_2_ NPs behave just like a soluble molecular acid with this *K*_eq_ as their *K*_a_, as supported by solvent isotope studies. Because the trap states have different compositions, their population and depopulation occur with the making and breaking of chemical bonds and not (as commonly assumed) just by the movement of electrons. In addition, the direct observation of a 2H^+^/1*e*^–^ trap state contradicts the emerging H atom transfer (1H^+^/1*e*^–^) paradigm for oxide/solution interfaces. Finally, this work emphasizes the importance of chemical stoichiometries, not just electronic energies, in understanding and directing the reactivity at solid/solution interfaces.

## Abstract 2

The two classes of trap states formed on reduction of colloidal TiO_2_ nanoparticles contain 1*e*^−^/1H^+^ and 1*e*^−^/2H^+^, differing by a proton, providing a chemical view of trap states in oxide materials.

## Introduction

Redox reactions of solids in contact with water are increasingly recognized to occur by proton-coupled electron transfer (PCET).^1−3^ In many but not all cases, the stoichiometry is close to 1*e*^–^ plus 1H^+^, for both semiconductor and metal interfaces. Similar chemistry is observed in aprotic organic solvents containing protic buffers,^4,5^ and for ion-coupled electron transfer in relatively proton “free” media, such as in lithium-ion batteries.^6^ This report connects the PCET stoichiometry of semiconducting oxides with the chemical nature of their surface states using colloidal titanium dioxide (TiO_2_) as an example.

**Scheme 1:**
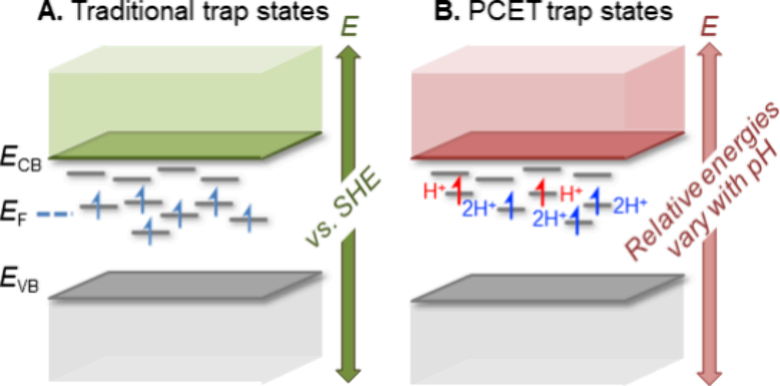
Electronic versus PCET Trap State Models

Semiconducting materials almost invariably have “trap states” that can be occupied by additional electrons or holes. Trap states are prevalent at interfaces or defects, where the material is not the same as the ideal bulk. For oxide semiconductors, modulation of defects (e.g., vacancies) to form “shallow” or “mid-gap” trap states can change the charge carrier density, electrocatalytic activity, or charge trapping and recombination ability of the material.^7−12^ Trap states are particularly important in photophysical, photochemical, and electrochemical processes for energy storage and conversion. To connect with knowledge established in solid-state physics, trap states are often considered to be electronic states with a smoothly varying density of states usually in the band gap of a material (Scheme 1A).^7,13−17^

For most materials, including TiO_2_, the *chemical* nature of the trap states remains obscure. Even for extensively studied luminescent quantum dots, “shallow” and “deep” trap states (with the energy relative to the conduction band) can only be correlated with undercoordinated surface ions and stacking faults.^18^ For oxide materials, molecular clusters may provide the best available atomistic models for trap states and other aperiodic defects.^19,20^

Herein, this report shows that different classes of trap states in 4 nm TiO_2_ nanoparticles (NPs) differ in their chemical composition by their number of protons (Scheme 1B). This case study shows that the population and depopulation of trap states can be coupled to the making and breaking of chemical bonds rather than being just the movement of electrons.

Reduction of TiO_2_ NPs has long been known to populate a variety of trap states, and studies have implicated an overall stoichiometry of 1H^+^ per 1*e*^–^.^3,21−27^ Our laboratory has found that a common preparation of aqueous TiO_2_ colloids^28^ gives NPs whose trap states fall into two distinct classes with unique EPR signatures (see below and Supporting Information (SI) Section S1.2). These trap states were named **Blue** and **Red** based on their different optical spectra and are in equilibrium.^25^ Kinetic studies showed that the **Red** trap states in the equilibrium are oxidized ∼5 times faster than the **Blue** ones.^29^

We report here that the **Blue**/**Red** equilibrium in these anatase TiO_2_ NP colloids is shifted by solution pH and solvent isotopologues (H_2_O vs D_2_O).^3,29^ The data demonstrate a 1H^+^ difference between the two states as well as their absolute stoichiometries. Our pH and spectroscopic results also showed these *nanoparticles* behave like *molecular* acids. Such a difference in chemical stoichiometry between trap states has rarely been discussed, but we suspect that it will prove to be common for semiconductors with redox-active surfaces. Understanding the chemical nature of trap states should facilitate trap state engineering. One can imagine that the ability to control and differentiate trap state reactivity could prove to be attractive in energy conversion and chemical production processes.

## Results and Discussion

A broad description of our experimental setups is presented here; additional details and control studies are further elaborated on in the Supporting Information (SI). As in prior studies, the TiO_2_ colloids were prepared by hydrolysis of TiCl_4_ in 18 MΩ water.^25,28^ The particles are mostly anatase, with an average diameter (TEM) of ∼4 nm; 1000 Ti atoms/NP based on previous studies.^25^ The colloids (3 mg TiO_2_/mL) had [Ti]_total_ = 28 mM (by ICP-MS), corresponding to 30 μM NPs, and they were pH ∼ 2.3 after dialysis. Reduced TiO_2_ colloids (15 mg/mL), TiO_2_^*R*^, were prepared by UV photolysis in the presence of 0.15 M methanol (as a sacrificial reductant) and allowed to thermally and chemically equilibrate (Figure S1).^3^ The TiO_2_^*R*^ colloids typically had an effective solution concentration of 12 ± 2 mM *e*^–^, determined by spectrophotometric titrations with aliquots of 4-MeO-TEMPO or KI_3_, following previous work (Figure S2).^3^ This corresponds to 40 electrons per nanoparticle (12 mM *e*^–^/150 μM NPs), or about 5–8% reduction of the Ti^4+^.

In a typical experiment, the stock TiO_2_^*R*^ NPs were divided into multiple batches, each then diluted 10-fold with 18 MΩ H_2_O (i.e., now 1.2 ± 0.2 mM [*e*^–^]) in the presence or absence of additional aqueous HCl or Me_4_N^+^OH^–^ (TMAOH; SI Section 3.1). [***CAUTION:*** Me_4_N^+^ (TMA) *is highly toxic* via absorption through the skin.^30,31^ We now avoid using it and recommend that readers use alternatives, e.g., NMe_3_Bz^+^OH^–^.]

The 10-fold diluted solutions without acid/base addition typically had a pH of ∼2.6–2.8, while the acidified/basified solutions were in the range of pH 2–3 (bulk [H^+^] = 10–1 mM). The colloids were kept at pH 2–3 due to particle instability outside this range. pH and spectroscopic (UV–visible, electron paramagnetic resonance [EPR]) studies were conducted on the same solutions for consistency. Previous studies have shown that freezing of the samples for EPR measurements did not affect the Red/Blue ratio (equilibration is slow).^25,29^ Parallel deuterium studies used the respective isotopologues (e.g., D_2_O, DCl). Before dilution, D_2_O and H_2_O were degassed to remove any ambient air (see SI Section 1.1). All measurements with TiO_2_^*R*^ were conducted in a nitrogen-filled, water-compatible glovebox. Due to inherent batch-to-batch variability, comparisons were made between samples from the same photolysis batch on the same day. Thus, the electron concentration and ratio of trap states were initially the same. This means all *relative* changes could be attributed to the postphotolysis treatment, and pH, UV–vis, and EPR results can be correlated at a specific pH as well as across the whole pH series. For instance, the titratable reducing equivalents in TiO_2_^*R*^ did not change as a function of pH (Figure S8).

### Changes in the Blue/Red Equilibrium with pH

**Figure 1:**
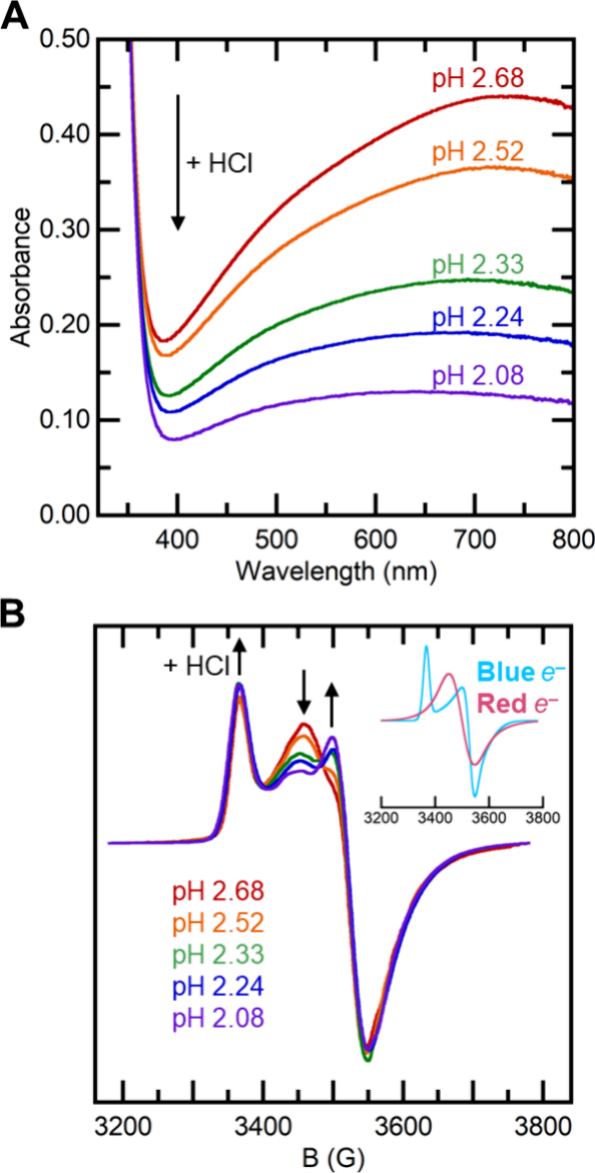
(A) Overlaid UV–visible spectra of TiO_2_^*R*^ after photolysis (pH 2.68, red trace) and with pH adjustments using HCl. The broad absorption decreased in intensity, and the λ_max_ blue-shifted at lower pH. (B) Overlay of EPR spectra (measured at 10 K, spin density normalized) of samples at different pHs. The inset shows the individual fitted spectra.^25,29^ The rhombic spectrum of **Blue** predominates at low pH, while at high pH the spectrum is closer to that of **Red**. See SI Section 4 for the EPR simulation details.

Addition of acid or base to TiO_2_^*R*^ changed their UV–visible spectra (Figure 1A; see Figure S4 for base addition). By eye, more acidic samples were a paler blue (Figure S3), which were corroborated spectrophotometrically by a blueshift in λ_max_ and a decrease in absorbance (Figure 1A). More basic samples showed the opposite changes, turning deep blue (Figure S4). The addition of acid to basified samples or base to acidified samples showed that this process was reversible (Figure S4). Control experiments adding the same concentration of counterions via potassium chloride (KCl) and tetramethylammonium chloride (TMACl [***CAUTION***]^30,31^) showed no spectroscopic changes, so the observed changes were due to protons and hydroxides, not to the solution ionic strengths (Figure S5).

EPR spectroscopy was also employed to characterize the electron populations over a pH range of 2.08 to 2.68, and a pH dependence was observed across the series. The spectra were double-integrated and normalized to the total spin density in each spectrum for ease of visual comparison (Figure 1B). All samples displayed EPR spectra that contained both a rhombic signal and an axial signal, as expected from prior studies.^22,25,29^ More acidic samples had more of the rhombic component (*g*_1_ = 1.990, *g*_2_ = 1.897, and *g*_3_ = 1.876), which is indicative of a higher **Blue** electron concentration. Samples at higher pHs had more of the axial component attributed to **Red** electrons (*g*_*⊥*_ = 1.922, *g*_||_ = 1.899). These results are consistent with the optical observations that the **Blue**/**Red** equilibrium is shifted toward **Blue** at lower pH.

The relative occupancies of **Blue** and **Red** states at equilibrium were best measured by simulating the EPR spectra in Figure 1B. As previously described,^29^ spectra were modeled using *g* and *g*Strain in EasySpin and letting the weights of the axial and rhombic contributions float freely (SI Section 4).^32^ Quantification of the **Blue**/**Red** ratio was less precise using broad, overlapping UV–visible spectra.

**Figure 2:**
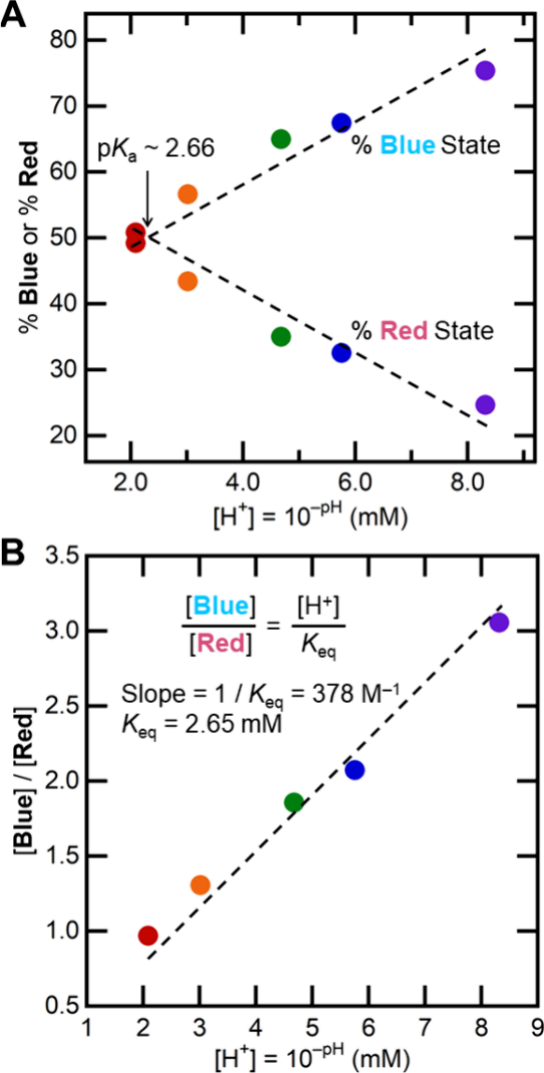
(A) Plot of %**Blue** and %**Red** electrons from the EPR samples in Figure 1B vs [H^+^]. Dashed lines are linear fits to %**Blue** or %**Red** versus [H^+^]. (B) Plot of the [**Blue**]/[**Red**] ratio vs [H^+^]. The slope of the line is 1/*K*_eq_, which is 2.65 mM (eq 2; the y-intercept is fixed at 0). Both plots used the percentages from EasySpin EPR simulations (SI Section 4).

The percentages of **Blue** and **Red** electrons varied directly with the solution proton concentration (Figure 2A). The equilibrium expression with **Blue** and **Red** differing by one proton would predict that the **Blue**/**Red** ratio is linear with [H^+^] (eqs 1 and 2; SI Section 5). The experimental data very closely fit eq 2 (Figure 2B). The inverse slope of the line in Figure 2B gives *K*_eq_ = 2.65 mM (Figure 2B). This value is in excellent agreement with the point in Figure 2A where %**Blue** = %**Red**: pH = p*K*_a_ = 2.66). eq 1 is also, by definition, the acid dissociation equilibrium of the **Blue** state, described by *K*_a_.
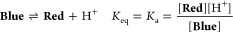
1
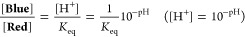
2

#### Chemically Reduced TiO_2_^R^

**Figure 3:**
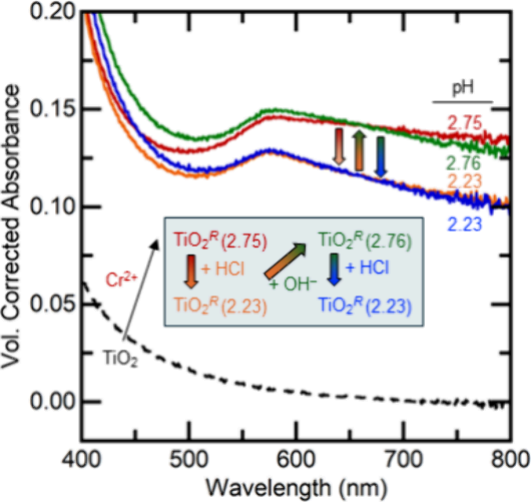
Optical spectra of TiO_2_ colloids (black dashed) upon the addition of Cr^2+^(aq) (red), then acid (orange), then base (green), and finally acid again (blue). The increase and decrease of the overall spectra show the shifting of eq 3 with pH, and the change in the shape of the spectra indicates the shifting of the **Blue**/**Red** equilibrium in eqs 1 and 2.

Chemical reduction of TiO_2_ was also explored to determine whether the **Blue**/**Red** equilibrium described above is related to the photoreduction of TiO_2_^*R*^. We had previously observed that aqueous Cr^2+^ formed an equilibrium with these colloidal TiO_2_, showing the same broad optical spectrum as for photoreduced TiO_2_^*R*^ (eq 3, Figure 3).^33,34^
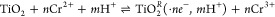
3Changing the pH of this equilibrium mixture with HCl and TMAOH has two effects (Figure 3). Addition of acid favors TiO_2_^*R*^ because reduction of TiO_2_ is proton-coupled, shifting eq 3 toward the right. Addition of a base reverses the changes. The more subtle effect of the acidification is the change in shape of the TiO_2_^*R*^ spectrum: the absorbance at 800 nm drops more than that at 600 nm. This is the same trend as was observed for photoreduced TiO_2_^*R*^ (Figure 1). These data are more difficult to analyze quantitatively because of the two effects and the absorbance from the chromium product(s). Still, the observation of the same reversible spectral response shows that the **Blue**/**Red** equilibrium in eqs 1 and 2 is independent of the method of formation of TiO_2_^*R*^.

### H^+^:e^–^ Stoichiometries for Blue and Red States

The previous section established that the **Blue** trap states contain one more proton than the **Red** ones, but it did not establish their stoichiometry relative to the starting, oxidized TiO_2_ NPs. Since most trap states are considered to be purely electronic states, it seemed reasonable that the **Red** states are formed solely by addition of electrons to TiO_2_, while the **Blue** states resulted from addition of 1*e*^–^ + 1H^+^. However, the results in this section show that colloids with mixtures of **Red** and **Blue** states liberate more than 1H^+^ per electron upon oxidation. The data indicate an *e*^–^: H^+^ stoichiometries of 1:1 for the **Red** states and 1:2 for the **Blue** ones.

**Figure 4:**
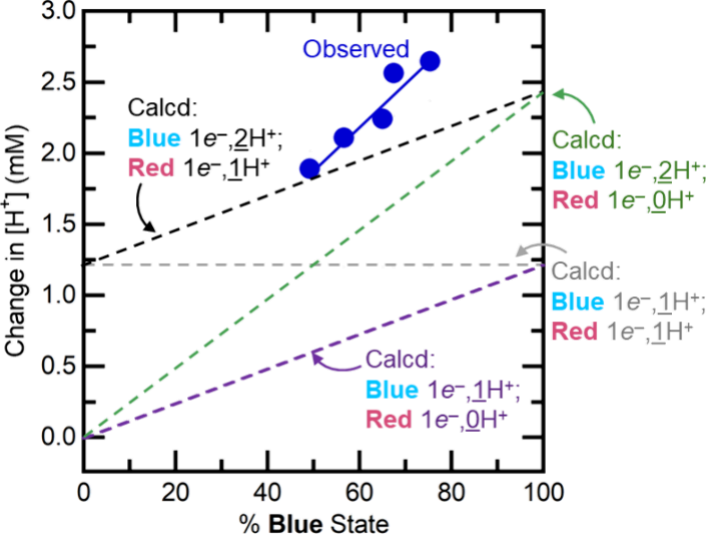
Amounts of protons liberated upon the oxidation of TiO_2_^*R*^ suspensions with KI_3_ (blue dots). Five aliquots of the same TiO_2_^*R*^ suspension (1.20 mM *e*^–^) were preadjusted to pH values between 2.08 and 2.68. The change in pH was measured for each aliquot and converted to the change in the H^+^ concentration (vertical axis). The %**Blue** (horizontal axis) was estimated from EPR (SI Section 4). Also included as dashed lines are the predictions of four models that assume integer **Blue**/**Red** H^+^ stoichiometries: 1:0 (purple), 1:1 (light gray), 2:1 (black), and 2:0 (green). The experimental Δ[H^+^] (blue circles) are closest to the model in which each **Blue** released 2H^+^ and each **Red** released 1H^+^ (black dashed; eqs 6, 7; see text). That conclusion is consistent with the 1H^+^ difference derived above. The deviation of the blue points from the predicted black dashed line is likely due to the model not including the change in the TiO_2_ buffer upon oxidation.

The proton stoichiometry was measured from the change in pH upon the oxidation of TiO_2_^*R*^ (eq 4). Five aliquots were taken from a TiO_2_^*R*^ suspension and adjusted to different pHs, and therefore different [**Blue**]/[**Red**] ratios. Each aliquot was oxidized with just enough KI_3_ to completely remove the 1.2 mM of trap state electrons, and the change in proton concentration was determined from the initial and final pH (eq 5; Figure S7, Table S2). KI_3_ was chosen as the oxidant because it and its product, iodide, have no pH-buffering activity. In each experiment, the pH decreased, showing the release of protons. Thus, proton release is coupled to electron removal.
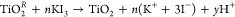
4
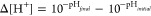
5The results of these five parallel experiments are plotted as the five dark blue circles in Figure 4. In each experiment, more than one proton was released per electron. The measured values range from 1.3H^+^/*e*^–^ for the aliquot initially with 49% **Blue**, to 2.3H^+^/*e*^–^ for the 75% **Blue** aliquot.

With the assumption of integer stoichiometries for each trap state, the results are most consistent with stoichiometries of 2H^+^ per **Blue***e*^–^ and 1H^+^ per **Red***e*^–^ (eqs 6 and 7). The prediction of this model is shown as a dashed black line in Figure 4.
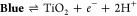
6
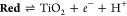
7The data are *not* consistent with the model where each electron in a **Blue** state is coupled to 1H^+^, while the formation of a **Red** state from oxidized TiO_2_ requires an electron, without proton coupling (the purple dashed line in Figure 4).

An alternative model with the **Blue** and **Red** states each having one proton per electron (light gray dashed line) does not fit the data and is not consistent with the conclusion above that **Blue** states have one more proton than **Red** states (eq 1).

The best fit line for the blue points in Figure 4 has a slope of 2.2 protons per electron in **Blue** and not in **Red**. This might suggest a 2:0 model where the **Blue** states have two protons per electron while the **Red** states are not proton-coupled. However, this model (purple dashed line) does not fit the data closely and does not obey the conclusion that **Blue** = **Red** + H^+^. Thus, the stoichiometries in eqs 5 and 6 are the best models for the data.

Still, the blue points in Figure 4 deviate from the 2H^+^ per **Blue***e*^–^ and 1H^+^ per **Red***e*^–^ model. The discrepancy is likely in part due to changes in the buffering of TiO_2_ upon oxidation, which is not included in the model.

### H^+^:*e*^–^ Stoichiometry, Equilibration, and Thermochemistry

**Scheme 2:**
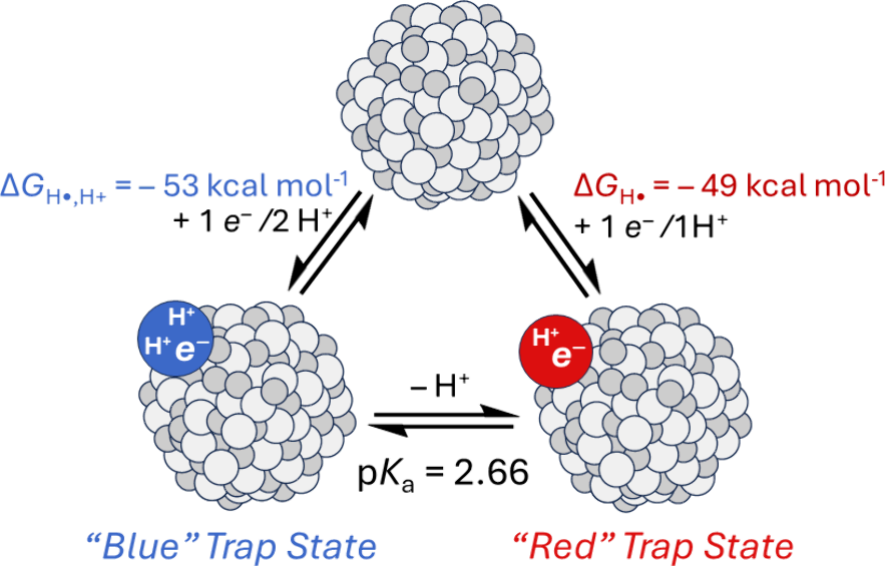
Thermodynamic Scheme of the Transfers of Electrons and Protons to Colloidal TiO_2_ NPs

The directly measured H^+^/*e*^–^ proton stoichiometries in Section 2 confirm the equilibrium derived from spectroscopic data in Section 1: each **Blue** state has one more proton than each **Red** state (eq 1 above). In addition, the data establish the absolute proton stoichiometry of both trap states (eqs 6, 7; Scheme 2). Both classes of trap states are stoichiometrically proton-coupled, contradicting the prevalent assumption that filling or emptying a trap state involves only the movement of an electron. Citrate-capped TiO_2_ have different trap states yet show similar behavior (SI Section 8), so this new paradigm may have some generality.

It was initially surprising that one class of trap states has a 2:1 H^+^:*e*^–^ stoichiometry. Most surface PCET reactions are thought to involve a 1:1 H^+^:*e*^–^ stoichiometry due to the energetic preference for charge balance. The addition of 2H^+^ + 1*e*^–^ increases the overall charge of the TiO_2_ NP by +1. The uncapped TiO_2_ NPs at pH 2 have a positive surface charge, as determined by zeta potential measurements,^3^ and the net charge is likely important in stabilizing the colloids in an aqueous environment. Both increasing the overall charge and increasing the average Ti oxidation state should increase the acidity of spectator TiOH and TiOH_2_ groups not directly involved in PCET. These effects likely contribute to the >1:1 H^+^:*e*^–^ stoichiometry of the **Blue** states. More generally, >1:1 H^+^:*e*^–^ stoichiometry, or “super-Nernstian” behavior, is increasingly observed for hydrous oxide materials. The **Blue** states perhaps provide a model for super-Nernstian behavior, which is poorly understood at the atomic level.^35−41^

Elucidation of the H^+^ stoichiometry allows us to build the thermodynamic relationships among oxidized, uncapped TiO_2_ NPs, the **Blue** state, and the **Red** state (Scheme 2). The free energy to add an H atom (1*e*^–^/1H^+^) to TiO_2_ to form the **Red** state, termed Δ*G*_H•_, is negative of the BDFE([TiO_2_]–H_***Red***_). The measured BDFE_***Red***_ of 49 kcal mol^–1^ is the same as the reported average [TiO_2_]–H BDFE for citrate-capped NPs, determined from measurements at pH 2–9.^3^

The thermochemistry of the **Blue** trap states cannot be described by a BDFE because they require the addition of 2H^+^ + 1*e*^–^ to TiO_2_. Since the p*K*_a_ of the **Blue** trap states is 2.66, these states are more stable than **Red** at the standard state (pH 0) by Δ*G*_PT_ = −3.62 kcal mol^–1^ (−1.36p*K*_a_). Therefore, the free energy to add H• + H^+^ to TiO_2_ to form a **Blue** state is the sum of the hydrogen atom and proton addition steps (eq 8).
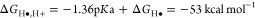
8The addition of H• + H^+^ is stoichiometrically the same as the addition of 2H^+^ + *e*^–^ or an H_2_^+^ ion. To our knowledge, the free energy to transfer >1:1 H^+^:*e*^–^ has not been quantified before, while those for hydrogen atom transfer (H•, 1:1 H^+^:*e*^–^) and hydride transfer (H^–^, 1:2 H^+^:*e*^–^) are well studied.^42,43^ We believe that such a thermochemical analysis for >1:1 H^+^:*e*^–^ super-Nernstian processes could be broadly valuable for hydrous oxides.^35−40^

The conclusion that both the **Blue** and the **Red** trap states are proton-coupled suggests that both are proton-accessible sites, likely near the NP surface. If both states are associated with the same small Ti_*x*_O_*y*_H_*z*_ region within each NP, their interconversion would require only the loss of a proton. However, if the states were spatially separated, conversion of **Blue** to **Red** would require proton loss coupled to movement of the electron from the region of the **Blue** state to that of the **Red** state on the time scale of the interconversion (∼5 min at room temperature).^29^ While the detailed movements of these small particles is still unclear, the PCET stoichiometries imply strong couplings of the H^+^ and *e*^–^, and suggest that they are relatively close.^42^

### H_2_O/D_2_O Equilibrium Solvent Isotope Effect (ESIE)

**Figure 5:**
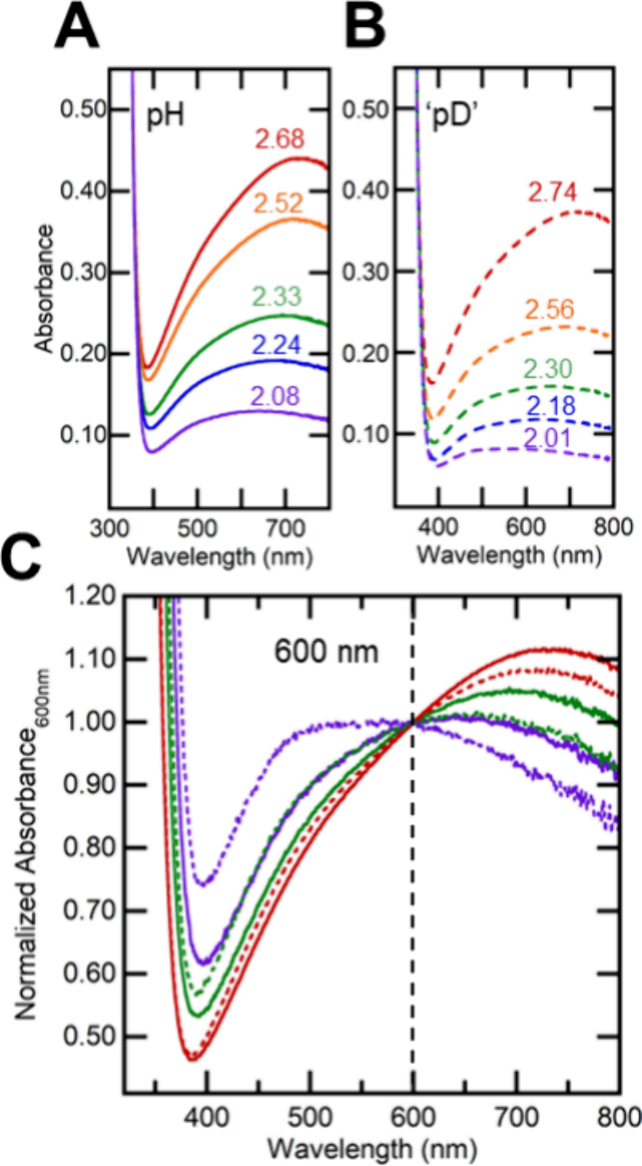
Optical spectra from addition of TiO_2_^*R*^ in pure H_2_O into a 9-fold excess of H_2_O+HCl (solid lines, pH) or 10%H_2_O/90%D_2_O+DCl (dashed lines, “pD”). The same line color indicates the same acid concentration ([HCl] = [DCl]; see workflow in Scheme S2). (A) Spectra of TiO_2_^*R*^ in H_2_O with the pH of each solution given in a colored number. (B) Spectra of TiO_2_^*R*^ in 10%H_2_O/90%D_2_O (dashed lines) with the “pD” of each solution from eq 9 given in a colored number. (C) Spectra from **A** and **B** overlaid and normalized to Absorbance_600 nm_ = 1. The shifts in λ_max_ and spectral shape show that the samples in H_2_O contain more **Red** than **Blue** than their counterparts in D_2_O, and that samples at higher pH (or “pD”) contain more **Red** than **Blue** than those at lower pH (or “pD”). The similarity of the overall spectral shapes indicates that the component **Red** and **Blue** spectra do not vary substantially between H_2_O and D_2_O.

The effect of changing the solvent from H_2_O to mostly D_2_O provides additional insight into the **Blue** and **Red** trap states. In one set of experiments, aliquots from the same TiO_2_^*R*^ suspension in H_2_O were diluted 10-fold into H_2_O or D_2_O. To our surprise, the two solutions were visibly quite different (see Figure 5A and B, and Scheme S2 with x = 0). The TiO_2_^*R*^ in 10% H_2_O/90% D_2_O (Figure 5B, red dashed) was paler in color, with lower absorbance and with a higher energy λ_max_, versus the TiO_2_^*R*^ in 100% H_2_O (red dashed vs red solid lines in Figure 5A vs B). These spectral changes indicated a shift of electrons from the **Red** to **Blue** states as the D_2_O content increased.

The spectrum of TiO_2_^*R*^ in 10% H_2_O/90% D_2_O was closely matched by a spectrum of TiO_2_^*R*^ in pure H_2_O that had been acidified from pH 2.68 to 2.52 with HCl (compare the red dashed and orange solid lines in Figure 5B and A, respectively). Since redox titrations of selected samples (diluted with H_2_O, H_2_O/HCl mixture, D_2_O, or D_2_O/DCl mixture) showed the same [*e*^–^] in all the samples (Figure S8), the spectral differences are solely due to the samples being 100% H vs 90%D/10%H. The spectra therefore show the presence of a significant equilibrium solvent isotope effect (ESIE).

To examine the ESIE, a series of TiO_2_^*R*^ samples were acidified by either HCl/H_2_O or DCl/D_2_O mixtures to form matched pairs of solutions (Scheme S2). The TiO_2_^*R*^ in D_2_O/DCl samples were consistently less absorbing and more blue-shifted than those in the same concentration of H_2_O/HCl (Figure 5A and B). To emphasize the changes in shape with the isotope and pH/pD, Figure 5C shows selected spectra normalized to their absorbance at 600 nm. Both the absolute absorbance and the change in shape show that the D_2_O samples had a shift in equilibrium from **Red** to **Blue** states as compared to H_2_O samples with the same concentration of HCl/DCl.

Quantitative interpretation of results from D_2_O/DCl and H_2_O/HCl experiments requires a way to compare the acidities of the isotopically different suspensions. The difficulty in comparing pH in H_2_O with pD in D_2_O has long been recognized as a challenge in ESIE measurements, in part because it is believed to be impossible to do rigorously. pH meters calibrated with standard buffers in H_2_O give lower values in analogous D_2_O solutions, termed pH*. The pD is typically estimated as the experimental pH* plus 0.40–0.45 to pH*,^44−47^ although this has been disputed for solutions far from pH 7.^48,49^ We have developed an empirical estimate of the pH*/pD correction factor at low pH in the presence of oxidized and buffering TiO_2_ NPs (details in SI Section 7; Figures S9, S10).^50−53^ The estimated correction is linear with the volume fraction of D_2_O, following eq 9, in which “pD” is used for any H_2_O/D_2_O mixture. For 10%H_2_O/90%D_2_O suspensions, the correction is 0.32.
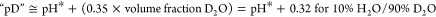
9

With this empirical pH/pD comparison, we return to the very similar solid-orange and dashed-red spectra in Figures 5A and B. The solid-orange colloid was at pH = 2.52 while the dashed-red one had a measured pH* of 2.42, which implies ‘pD’ = 2.74. Thus, to get the same **Blue**/**Red** ratio, *the TiO*_*2*_^*R*^*in D*_*2*_*O must be more basic (less acidic) than in H*_*2*_*O*. In other words, the **Blue** state is less acidic in D_2_O than in H_2_O so that a higher pD is needed to have the same **Blue**/**Red** ratio. This higher p*K*_a_ of the **Blue** state is the origin of ESIE observed in the spectra.

The lower acidity of the **Blue** trap state in D_2_O parallels the behavior of *molecular* acids. Many studies of H_2_O/D_2_O ESIEs for molecular acids conclude that DA in D_2_O is a weaker acid than HA in H_2_O.^54−57^ At lower pHs, the difference is typically a factor of 3, or Δp*K*_a_ = p*K*_a_^D2O^ – p*K*_a_^H2O^ ≈ 0.5 (or a factor of 2.7 and Δp*K*_a_ ≈ 0.45 in 10:90 H_2_O:D_2_O).^54,57^ This is in part due to the stronger solvation of ions in H_2_O, as evidenced by the larger autoprotolysis constant of H_2_O and the higher heats of solution for ionic compounds in H_2_O.^58,59^ The spectral changes observed here suggest that **Blue** is about 2 times weaker in D_2_O than in H_2_O (Δp*K*_a_ ≈ 0.3). Deprotonation of **Blue** decreases the positive charge on the TiO_2_ NPs which is less favored in D_2_O.

Thus, the **Blue** trap state acts as a simple monoprotic acid, as shown in eq 1 above. The case study in this paper shows that the physical organic approach long used for small molecule analysis can be extended to (nano)materials such as TiO_2_ NPs. From the physical-organic perspective, a full description of semiconductor trap states must, of course, start with any changes in chemical stoichiometry.

## Conclusions

Equilibration and ΔpH studies show that the two classes of electron trap states in reduced colloidal TiO_2_ NPs are both proton-coupled and that they have different proton stoichiometries. Electron addition to a “**Red**” trap state occurs with the uptake of one proton from solution. Reduction to “**Blue**” states involves *two* protons. The interconversion of these two classes of states involves solely a proton and no net electron change. This interconversion follows the simple equilibrium expression **Blue** ⇌ **Red** + H^+^ (eq 1 above), whose equilibrium constant is by definition the acid dissociation constant (*K*_a_) of the **Blue** trap states. The H_2_O/D_2_O solvent equilibrium isotope effect on this *K*_a_ supports strong analogy with soluble molecular acids.

The results reported here challenge the typical interpretation of trap states in which their population and reactivity involve solely transfer of an electron. In these TiO_2_ NPs, changes in trap state occupancy involve transfer of one or two protons in addition to an electron. These results prompt a re-examination of the description and reactivity of trap states; for example, the traditional treatment with a Fermi–Dirac distribution of pure electronic states is not appropriate.

This study was possible because these TiO_2_ NPs have two and only two distinguishable classes of trap states. Typically, oxide semiconductors have many types of trap states, preventing quantitative analysis. We believe that many other systems likely have trap states that change their composition upon population, but to our knowledge, this is the first report of such behavior.

These results also challenge the emerging PCET paradigm of ideal 1H^+^:1*e*^–^ stoichiometry at materials. The two-proton per electron stoichiometry of the **Blue** trap states is, to our knowledge, unprecedented in materials chemistry. The presence of multiple trap states with different proton stoichiometries provides a possible explanation of the super-Nernstian behavior (>60 mV/pH) increasingly observed for hydrated oxides with valuable properties.^41^

The chemical reactivity of trap states is particularly important for emerging energy conversion technologies. The trap states in the TiO_2_ NPs reported here are isoergonic at pH 2.7, differ by a single proton, and have a 5-fold difference in their rate constant of oxidation by TEMPO.^29^ These results suggest the potential for trap state engineering to tune trap state properties for different applications. Additional studies are needed to test the generality of the conclusions from this one system. However, it is clear that the electron-focused or H atom-focused models are incomplete for this solid/solution interface. These trap states are not just buckets for electrons or holes.

## Supporting Information Available

General considerations regarding the reagents used as well as the synthesis and photochemical reduction of the TiO_2_ NPs; instrumentation details; general considerations for the reactions outlined in the main text; additional details on the spectrophotometric titrations of reduced TiO_2_, optical changes of TiO_2_ with pH, and EPR spectra and their simulations; additional experiments related to the proton stoichiometry, titrations with KI_3_, and D_2_O (pD) correction factors; EPR and UV–vis data for citrate-capped NPs; controlled studies with formic acid/formate salt addition to TiO_2_^*R*^ (PDF)
Transparent Peer Review report available (PDF)
