# Supplementary material for: Trap States in Reduced Colloidal Titanium Dioxide Nanoparticles Have Different Proton Stoichiometries
Source: ACS Cent Sci. 2024 Nov 22;10(12):2266–73. doi: 10.1021/acscentsci.4c01074 (PMC11672544; doi:10.1021/acscentsci.4c01074)
Supplement: Supplementary file 1 — oc4c01074_si_001.pdf [file oc4c01074_si_001.pdf]

Supporting Information

Trap States in Reduced Colloidal Titanium Dioxide Nanoparticles Have Different Proton Stoichiometries

Noreen E. Gentry, Noah J. Gibson, Justin L. Lee, Jennifer L. Peper, James M. Mayer*

Department of Chemistry, Yale University, New Haven, Connecticut 06520-8107, United States.

Table Of Contents

1. General Considerations ... 2
1.1 REAGENTS ... 2
1.2 TiO_2 NANOPARTICLE SYNTHESIS AND PHOTOCHEMICAL REDUCTION ... 2
1.3 GENERAL CONSIDERATIONS FOR REACTIONS OF TiO_2^R ... 3
1.4 INSTRUMENTATION ... 3
1.4.1 Optical Spectroscopy ... 3
1.4.2 EPR Spectroscopy ... 3
1.4.3 pH Measurements ... 4
2. Electron Quantification by Spectrophotometric Titrations ... 4
3. Optical Changes with TiO_2^R ... 5
3.1 REVERSIBLE CHANGES WITH PH ... 5
4. EPR of TiO_2^R Colloids from pH 2.68 – 2.08 and pH* 2.42 – 1.69 ... 8
5. Delta pH Measurements and Calculations After Oxidation with KI_3 ... 10
6. H^+:e^- Stoichiometries for Red and Blue States ... 11
7. pH of the TiO_2 NP Colloids ... 12
7.1 PH AND PROTON CONCENTRATIONS ... 12
7.2 TiO_2 PH IN MIXED H_2O AND D_2O SOLVENTS ... 12
7.3 ACID TITRATIONS IN H_2O AND D_2O ... 14
8. Trap State Behavior of Citrate-Capped TiO_2 (*c*-TiO_2) ... 15
9. Effect of Formic Acid/Formate on Trap State Proton Equlibrium of TiO_2^R ... 16
10. References for Supporting Information ... 18

1. General Considerations

1.1 Reagents

18 MΩ·cm water (H_2O) from a Synergy® Milli-Q system was used in all cases. All reagents were used as received from Sigma Aldrich unless otherwise stated. 4-methoxy-2,2,6,6-tetramethyl-1-piperidinyloxy (4-MeO-TEMPO, Alfa Aesar, 98%+) and I_2 (Sigma-Aldrich, >99.8%) were purified via sublimation. Titanium tetrachloride ($TiCl_4$, Acros, 99.9%) was used as recieved.[1] D_2O was 99.9 atom% D (Sigma-Aldrich). H_2O and D_2O were sparged under N_2 at > 1 mL/min prior to use. Potassium triiodide (KI_3) solutions were prepared from I_2 and KI (Sigma Aldrich, 99%) in a 1:3 ratio by mass. Citric acid monohydrate (99%) was purchased from Fischer Scientific.

HCl (37% in H_2O, Sigma Aldrich) and tetramethylammonium hydroxide pentahydrate (TMAOH, Alfa Aesar) were diluted in H_2O to form 80 mM solutions for titrations. ***Caution! TMAOH is highly toxic** and is absorbed through the skin. It should be avoided when possible and only used with great caution.* See, for instance, Lin, C.-C.; Yang, C.-C.; Ger, J.; Deng, J.F.; Hung, D.-Z., Tetramethylammonium hydroxide poisoning. *Clinical Toxicology* **2010**, 48, 213-217, DOI: 10.3109/15563651003627777 and https://ehrs.upenn.edu/health-safety/lab-safety/chemical-hygiene-plan/fact-sheets/fact-sheet-tmah-tetramethylammonium (accessed 1/11/2024). If we were to perform these studies again, we would use benzyltrimethylammonium hydroxide solution (40 wt.% in H_2O, Thomas Scientific). Titrations with DCl were performed with an 80 mM solution prepared from 35 wt% DCl solution in D_2O (99 atom %D, Sigma-Aldrich).

1.2 TiO_2 Nanoparticle Synthesis and Photochemical Reduction

Anatase titanium dioxide nanoparticles (4 ± 1 nm) were synthesized as previously reported.[1-3] In short, $TiCl_4$ was hydrolyzed with Millipore water and then dialyzed in Millipore water until the solution reached pH ~2.3. Colloids were dried at 25 °C under the specific reduced pressure of 20 mTorr and resuspended into Millipore water at 3 mg/mL, unless otherwise noted (30 μM NPs, 1000 Ti atoms / NP based on previous studies[4]). The TiO_2 NPs were stable to agglomeration between pH 2 – 3 (likely due to their positive electrostatic charge).[1] Solutions of as-prepared colloids (oxidized) were clear and colorless (Figure S1A, left), with a flat, close-to zero absorbance across the visible spectrum above the band gap absorption (Figure S1B, black).

Citrate-capped TiO_2 (*c*-TiO_2) were prepared from the regular uncapped NPs following a previously reported procedure.[1] Briefly, uncapped TiO_2 NPs (pH ~2.3) were synthesized following the above procedure. The addition of citric acid monohydrate (4-20 mM) resulted in precipitation of a white powder and decrease in pH. Drop-wise addition of a concentrated, aqueous solution of TMAOH resulted in a resuspension of the NPs under rapid stirring.

The aqueous TiO_2 NP colloids were reduced to TiO_2^R via anaerobic UV photolysis, following reported procedures.[2-8] Samples were prepared in quartz windowed glassware (methanol was added to reach 0.15 M CH_3OH) and were degassed under nitrogen. Photolysis for typically 5-10 hours with a 100W Hg lamp (XLC, Inc.) with rapid stirring turned the solutions visibly blue (Figure S1A, right). As the NPs became more reduced, the overall absorbance increased and the λ_{max} redshifted (Figure S1B, purple to red). Reduced colloids (TiO_2^R) were thermally equilibrated in a nitrogen atmosphere (typically overnight) before use.

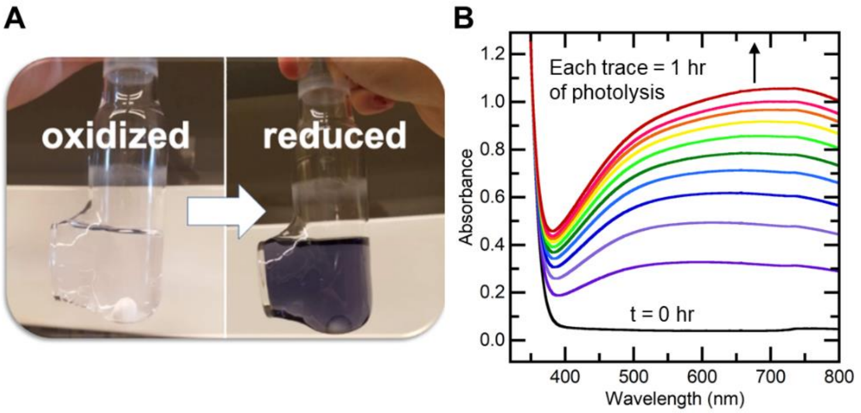

Figure S1 (**A**) Photographs of the oxidized (colorless, left) and reduced (blue, right) TiO_2 colloids. (**B**) UV-visible spectra of TiO_2 photolysis over 10 hours with 0.15 M MeOH. Bold lines show the optical spectra at 1-hour intervals.

1.3 General considerations for reactions of TiO_2^R

TiO_2^R colloids were manipulated in a nitrogen-filled, oxygen-free glove box set up to work with aqueous solutions. Oxidizing equivalents (μmol) required to quench all the photochemically-added electrons are used to calculate the concentration of TiO_2^R (mM). Photolysis for ~5 hours typically resulted in 1–1.5 mM e^- (~5% of the 28 mM [Ti] reduced; 150 mM MeOH as the sacrificial reductant is in large excess). The bulk proton concentration was determined using the solution pH, which was measured with a pH electrode ($\mathrm{pH} = -\log_{10}[\alpha_{\mathrm{H^+}}] \approx -\log_{10}[\mathrm{H^+}]$).

1.4 Instrumentation

1.4.1 Optical Spectroscopy

All optical spectra were collected using a Cary 5000 spectrometer with a scanning monochromator connected to a nitrogen-filled glovebox using Ocean Optics fiber optic cables. 1 cm-path length quartz cuvettes were used.

1.4.2 EPR Spectroscopy

EPR spectra were obtained with a Bruker EleXsys EPR Spectrometer equipped with a perpendicular-mode microwave cavity (4122 SHQE resonator) and a ColdEdge closed-cycle cryostat (Bruker) at 10 ± 0.1 K. Samples were flash frozen as described below and were all collected at 10 K. Typical parameters were as follows: microwave frequency, 9.38 ± 0.01 GHz; microwave power, 60 – 200 μW; modulation frequency, 100 kHz; modulation amplitude, 10.0 G; sweep time, 42.0 s; conversion time, 41.0 ms; time constant, 81.9 ms.

The EPR freezing process was delicate. It has previously been shown there is a temperature dependence on the **Red** and **Blue** equilibrium.[2] Thus, a slow freezing procedure would artificially increase the population of **Blue** relative to its room temperature distribution. However, as the solvent in these systems is primarily water, care was taken to avoid cracking the EPR tubes from the expansion of water. EPR samples were all prepared in a nitrogen-filled glovebox, where roughly 200 μL of sample were added to an EPR tube. The tube was capped

with a septum, sealed with electrical tape, and removed from the box. Within 5 minutes of removal, samples were flash frozen in a – 78 º C bath (dry ice and acetone). The bottom of the tube was first immersed into the cold bath in a quick up and down fashion. As the bottom portion froze, more of the tube was allowed to go into the cold bath on the downward motions until the entire sample was frozen. Once frozen, the samples were moved to liquid nitrogen, where they were stored until the EPR measurement.

1.4.3 pH Measurements

pH measurements were collected using a Thermo Scientific™ Orion™ PerpHecT™ ROSS™ Combination pH Micro Electrode inside the glove box, calibrated using five NIST pH standard solutions in water (H_2O) from BrandNu: 1.68, 4.01, 7.00, 10.01, 12.46.

2. Electron Quantification by Spectrophotometric Titrations

Batches of TiO_2 NPs were prepared following the procedure in section S1.2. These NPs were photolyzed for ~5 hours and the final pH of the TiO_2^R was recorded to be 2.68. To determine the average number of electrons in the NP batches, titrations with 4-MeO-TEMPO radical, a 1 $e^–$:1 H^+ acceptor, and KI_3, a 2 $e^–$ acceptor (per $I_3^–$), were performed under an inert atmosphere. The change in absorbance was monitored by UV-vis spectroscopy and an end point was reached when no change was observed with additional aliquots of the oxidant (Figure S2).

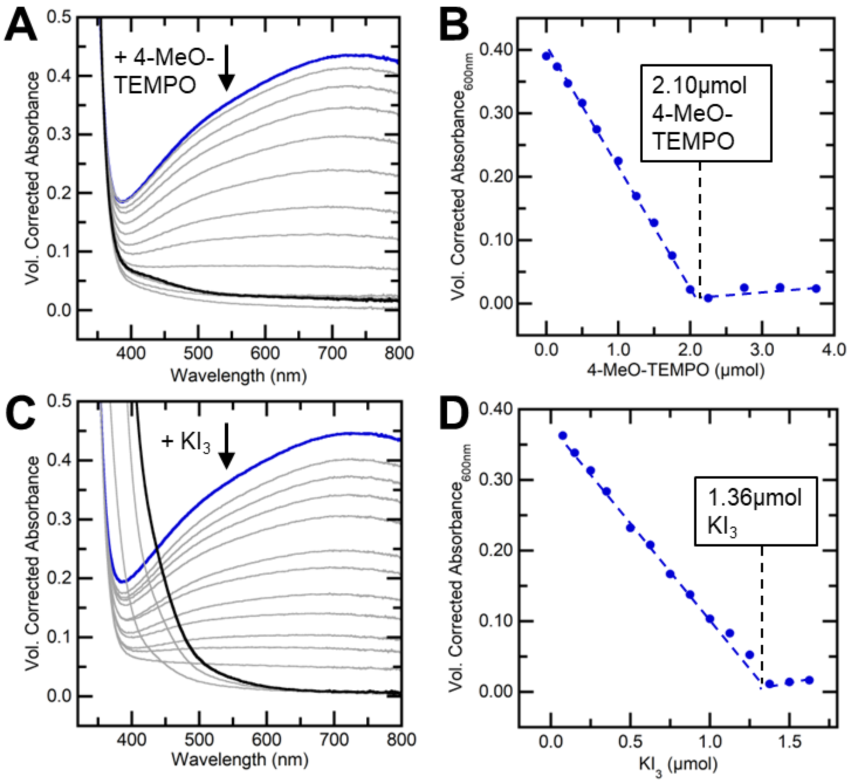

Figure S2 (**A**) Spectrophotometric titration of TiO_2^R NPs (pH 2.68) with 4-MeO-TEMPO radical. The blue trace is the initial spectrum before 4-MeO-TEMPO addition. The black trace is after the final aliquot. (**B**) The volume corrected absorbance at 600 nm versus the μmoles of 4-MeO-TEMPO added to the TiO_2^R NPs with linear fits before and after

the endpoint. The endpoint (2.10 μmol 4-MeO-TEMPO) is marked by the black dashed line. (**C**) This process was repeated using KI_3 on the same batch of NPs. The blue trace is the initial spectrum and the black is the final spectrum after addition of KI_3. The increase in absorbance at wavelengths < 500 nm is due to excess KI_3. (**D**) The volume corrected absorbance at 600 nm versus the μmoles of KI_3 added to the TiO_2^R NPs with linear fits before and after the endpoint. The endpoint was found to be 1.36 μmol of KI_3.

The spectrophotometric titrations using KI_3 and 4-MeO-TEMPO had similar endpoints (1.36 μmol KI_3 and 2.10 μmol 4-MeO-TEMPO). The final electron concentration [e^-] was taken as the average of both endpoints which was found to be 1.2 ± 0.2 mM e^- similar to previously reported batches.[1-2,4]

3. Optical Changes with TiO_2^R

The addition of acid or base to TiO_2^R led to systematic changes in the optical and chemical properties of the colloids. UV-visible spectroscopy was used to track these changes *post*-photolysis. In addition to spectroscopic changes, some of these changes were large enough to distinguish by eye. Shown below is an example of the color change in samples ranging from pH 2.19 – 2.84 in colloids that had 0.91 mM e^-. Going from left to right, the samples became less absorbing and paler as the pH was lowered (Figure S3).

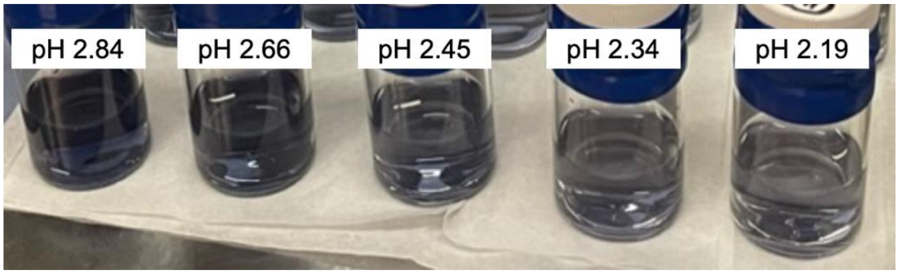

Figure S3. Photograph of solutions of TiO_2^R (0.95 mM e^-) that have been adjusted to different pHs *post*-photolysis, resulting in a visible color change. Samples that are less acidic are more absorbing and deeper blue in color, corresponding to more **Red** states.

3.1 Reversible changes with pH

Optical experiments were performed to assess the reversibility of the spectroscopic changes associated with pH change. A stock solution of TiO_2^R (0.87 mM e^-, pH 2.43) was split into two batches, and tetramethylammonium hydroxide (TMAOH; see *Caution* above) or hydrochloric acid (HCl) was systematically added into each sample to change the pH (Scheme S1). Once each sample neared the limits of colloid stability (pH ~ 2 and pH ~ 3), acid was added to the basic sample (Figure S4A, B) and base was added to the acid sample (Figure S4C, D). Optical spectra were recorded of each sample and showed the overall spectra were maintained throughout this process. The change in absorbance at 600nm plotted against the change in pH showed good reversibility with acid-base addition.

Scheme S1 Experimental design for the reversibility of optical changes as a function of pH.

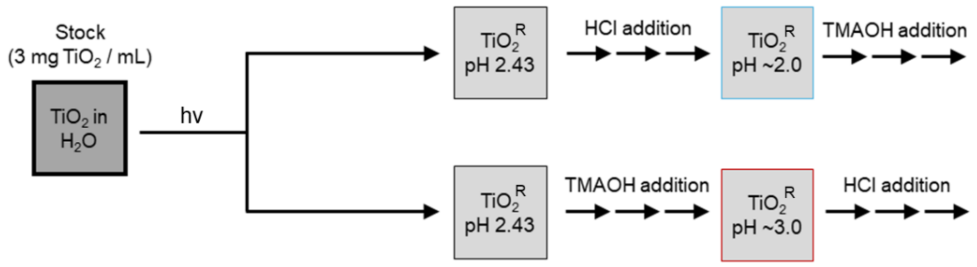

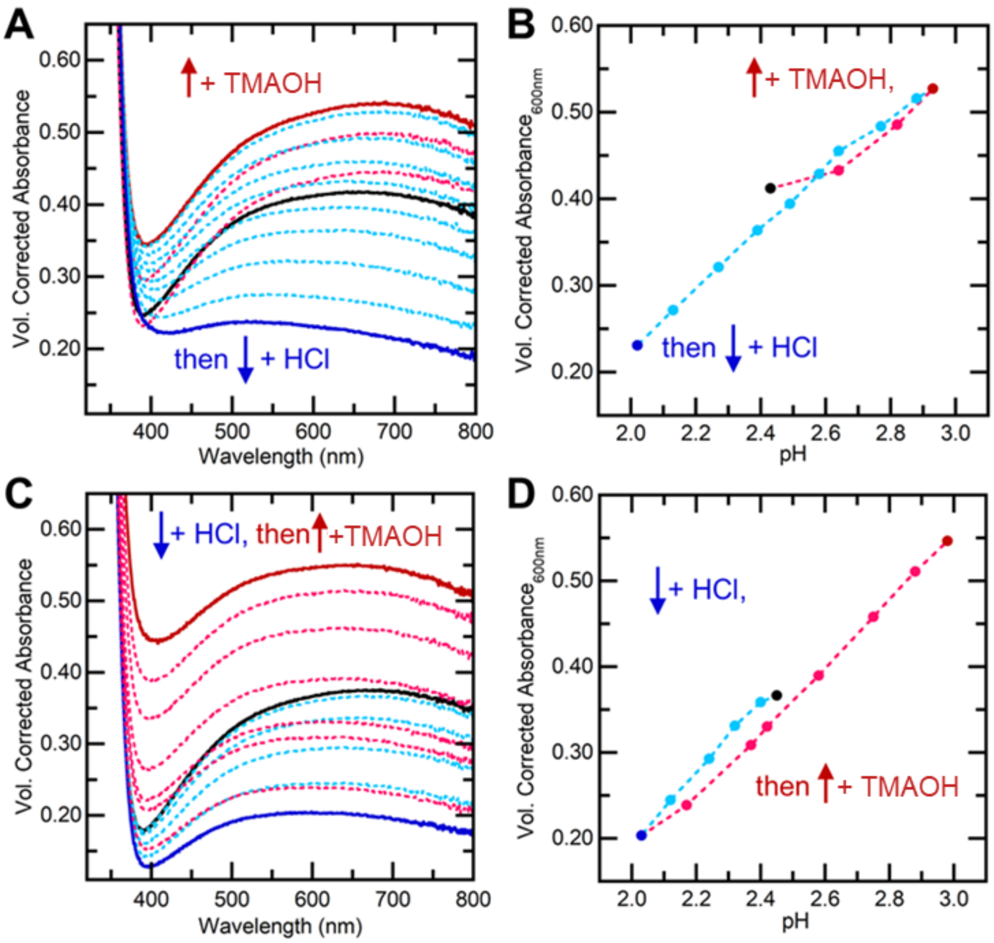

Figure S4 (**A**) Optical spectra of TiO_2^R (0.87 mM e^-, pH 2.43) upon the addition of TMAOH (see *Caution* above) up to pH 2.93 (black to red traces) and the return spectra with acid addition down to pH 2.02 (turquoise to blue). (**B**) Plot of volume corrected absorbance at 600 nm versus pH after TMAOH then HCl additions. (**C**) Matched cuvette to (A) where the equivalent concentration of HCl was added down to pH 2.03 (black to blue trace) and TMAOH was added to pH 2.98 (pink to red trace). (**D**) Plot of volume-corrected absorbance at 600 nm versus pH after HCl then TMAOH additions.

Complementary experiments with the same concentrations of counterions (TMACl [see *Caution* above] or KCl) did not lead to the same dramatic changes in the optical spectra (Figure S5). Compared to addition of acid and base, this reiterates the lack of ionic influence on these observations.

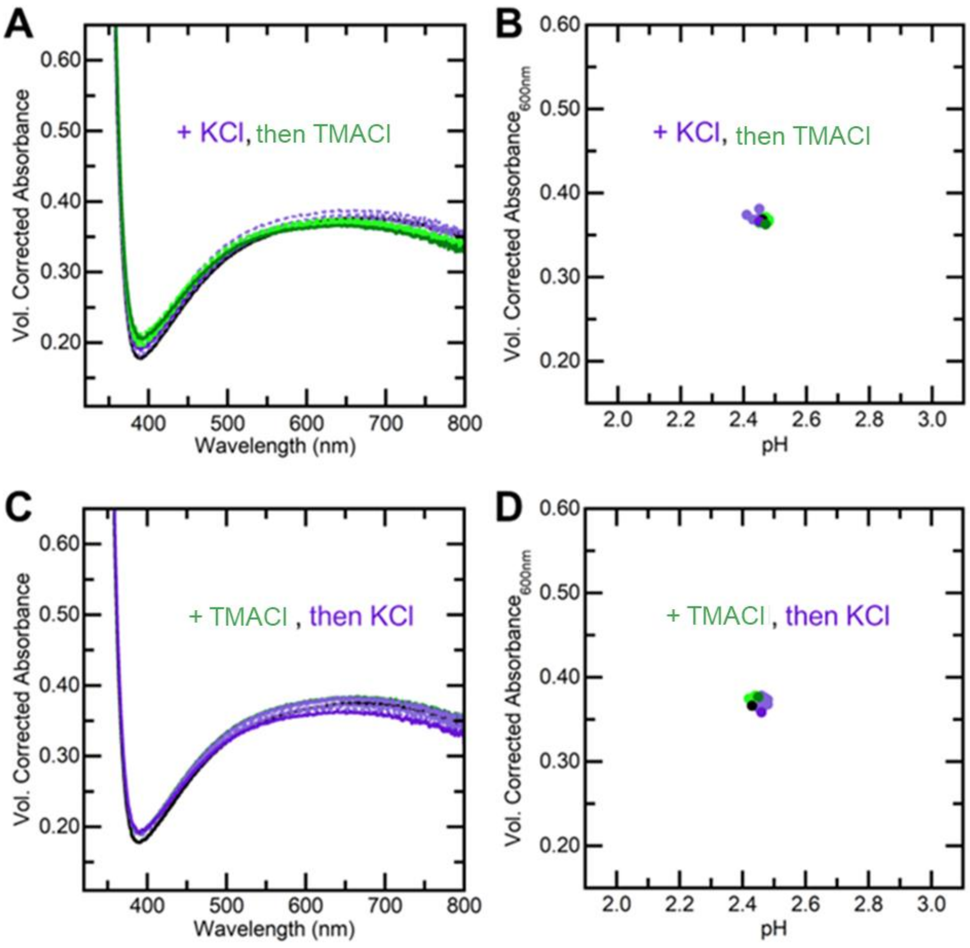

Figure S5 (**A**) Optical spectra of TiO_2^R (0.87 mM e^-, pH 2.43) upon addition of KCl then TMACl, equivalent to HCl and TMAOH added in Figure S4A and B. (**B**) Volume corrected absorbance versus pH after KCl and TMACl additions. (**C**) Equivalent cuvette to (A) with addition of TMACl (see *Caution* above) followed by KCl. (**D**) Volume corrected absorbance versus pH with TMACl and KCl additions. Negligible pH or absorbance changes were observed in either experiment.

We also explored alternative avenues to generate TiO_2^R NPs other than photoreduction. We found Cr^{2+} in an acidic medium (pH ~ 2) can partially reduce TiO_2 to TiO_2^R. In Figure 3, addition of 2 µmol $CrCl_2$ to a TiO_2 suspension in degassed water (3 mg/mL; black dashed trace) generated TiO_2^R (red solid trace) at pH 2.75. Optical experiments were performed to assess the reversibility of the spectroscopic changes associated with pH change. 80 mM stock solutions of HCl and TMAOH (see *Caution* above) were prepared. Adjustment of the TiO_2^R solution to pH 2.23 using acid resulted in the orange trace, which absorbance (especially at ~800 nm) was lowered. Addition of base to this solution restored the pH to 2.76 and the optical spectrum approximately returned to its original absorbance (green trace). Subsequent addition of acid to adjust the pH to 2.23 yielded the blue trace, which overlays well with the orange spectrum (also at pH 2.23). This series of experiments demonstrated the trap state proton equilibrium of TiO_2^R generated *chemically* is similar to that generated *photochemically*.

4. EPR of TiO_2^R Colloids from pH 2.68 – 2.08 and pH* 2.42 – 1.69

To examine the **Red : Blue** ratios under different pH and pH* conditions, a concentrated solution of TiO_2^R was prepared with an initial pH of 2.02 and 12 ± 2 mM e^-. An aliquot of this stock solution was diluted 10-fold into D_2O or H_2O before being titrated with HCl and DCl to expand the pH range (Scheme S2). Samples at different pHs at ambient temperatures were flash frozen as described in section S1.4.2 and EPR spectra were collected at 10 K.

Scheme S2. Standard dilution procedure for isotope experiments

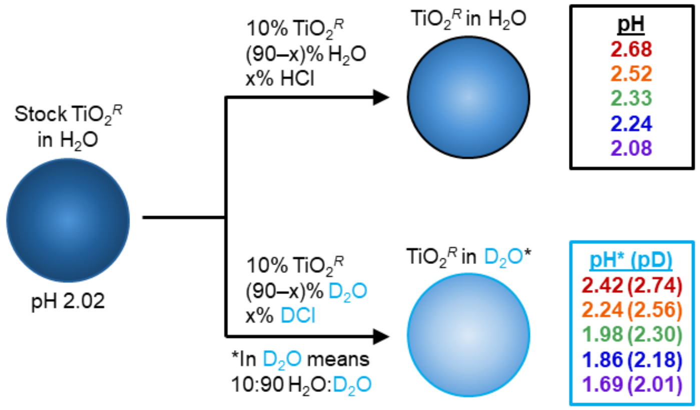

The EPR spectra (black solid traces) are presented in Figure S6. They show the general trend that as the sample pH decreased, the rhombic contributions (blue dashed) increased and axial (red dashed) decreased. To allow for ease of shape comparisons, samples were normalized by their total spin density (the double integral of each spectrum). The purple dashed traces are the overall simulated fits to the experimental spectra (black solid).

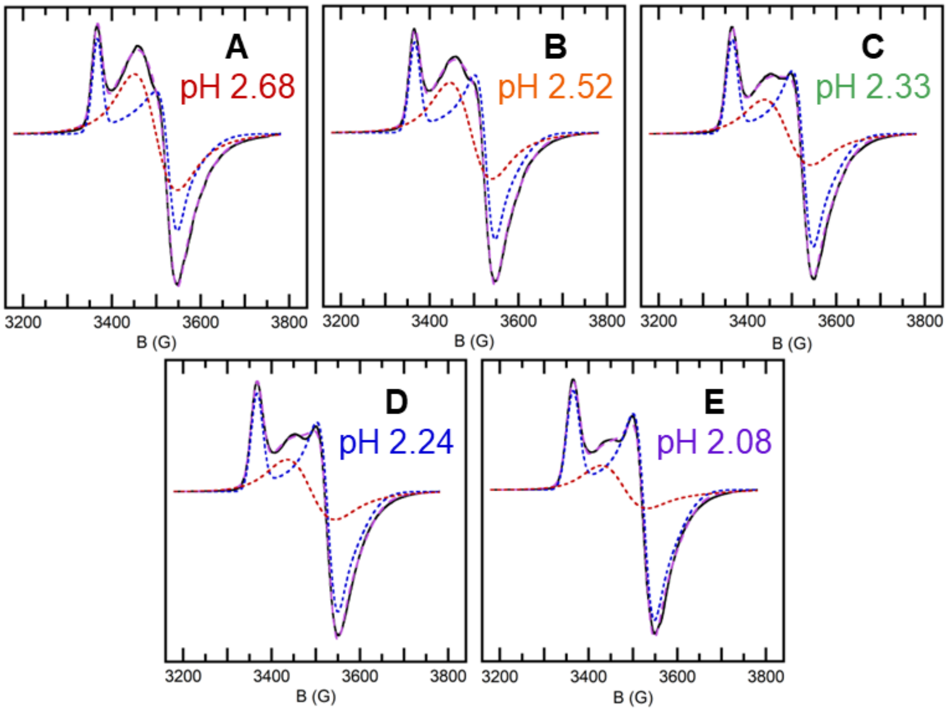

Figure S6 (**A-E**) EPR spectra for pH 2.68-2.08 TiO_2^R (1.2 ± 0.2 mM e^-) at ~10K prepared by dilution in H_2O and then acidified with HCl. The black trace is the experimental data. The red and blue dashed traces are the modeled axial and rhombic components from the EasySpin pepper function corresponding to the **Red** and **Blue** states, respectively. The sum of the two trap state fits is the dashed purple trace in each spectrum.

All EPR spectra were simulated using the EasySpin pepper function[9] for solid state (frozen) samples, as was done in previous publications with these TiO_2^R colloids.[2,4] Each spectrum was simulated as a sum of two spin components, an axial and a rhombic signal, with line broadening modeled using *g*Strain. The simulation was optimized using a Nelder/Mead downhill simplex least-square fitting esfit EasySpin function. Using this optimized model, all samples were simulated with minimal flexibility permitted in their *g* and *g*Strain values. This was done to maintain a consistent model across samples to better encapsulate any broadening or slight shifts associated with pH change. The rhombic and axial signal weights were allowed to float freely, and these values were used to determine the relative **Blue** (rhombic) to **Red** (axial) ratios. The average parameters for these simulations are given in Table S1 and the **%Blue** can be found in Table S2 in section SI-5.

Table S1 Parameters used to model EPR samples presented in Figure S6.

	H_2O series		D_2O series	
Component	*g*	gStrain	*g*	gStrain
Axial (Red)	1.922±0.004	0.053±0.003	1.934±0.007	0.051±0.007
	1.899±0.010	0.150±0.003	1.875±0.019	0.129±0.035
Rhombic (Blue)	1.990±0.001	0.017±0.002	1.990±0.001	0.017±0.001
	1.897±0.001	0.019±0.001	1.898±0.001	0.019±0.001
	1.876±0.002	0.059±0.005	1.872±0.002	0.052±0.002

5. Delta pH Measurements and Calculations After Oxidation with KI_3

The oxidation of TiO_2^R with an electron transfer-only reagent resulted in an increase in the acidity of the solution. A solution of potassium triiodide was prepared by first dissolving 15 mM potassium iodide (KI) in solution. Next, 5 mM iodide (I_2) was added and the solution turned dark red. The solution was stirred for several hours until the I_2 was fully solubilized.

A stock solution of concentrated TiO_2^R was diluted 10-fold into H_2O, resulting in a pH of 2.68. This solution was spectrophotometrically titrated with KI_3, giving an electron concentration of 1.2 ± 0.2 mM. The stock solution was 10-fold diluted into four more HCl/H_2O mixtures to expand the pH range from 2.08 – 2.68. Each sample was reacted with 1.38 μmol of KI_3 to fully oxidize the electrons and liberate any coupled protons. The optical spectra were recorded before and after this oxidation, and then the pH of the oxidized samples was recorded (Figure S7).

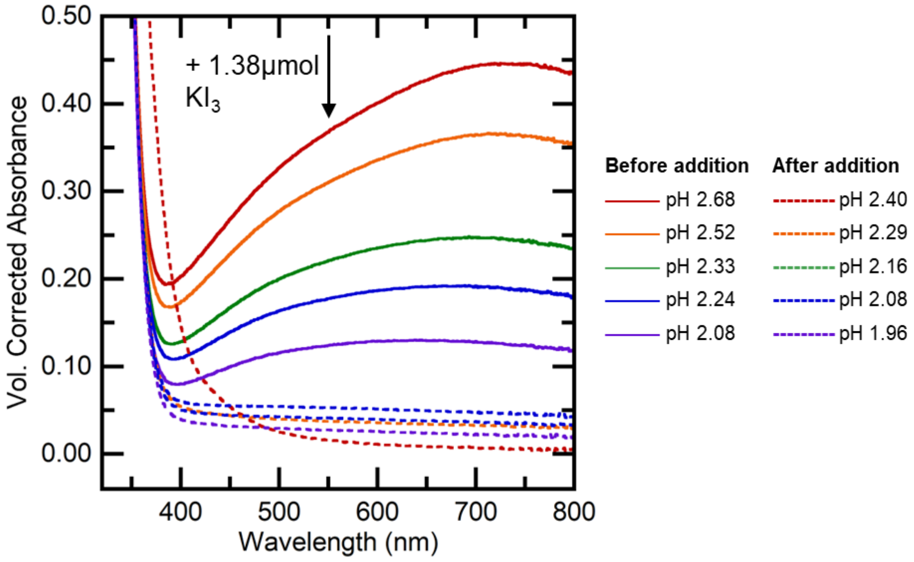

Figure S7. Spectra of TiO_2^R colloids (1.2 ± 0.2 mM e^-, solid traces) at different pH from 2.08 – 2.68. Dashed traces show the spectra after the addition of 1.38 μmol of KI_3.

Using the electron concentration (1.2 ± 0.2 mM) and the **%Red** and **%Blue** at different pHs from EPR, the **Red** and **Blue** concentrations in each sample can be calculated. It was determined **Red** and **Blue** differ in composition by one proton (see the main text), and since these electrons have been shown to be proton-coupled, it was hypothesized **Blue** is coupled with $2H^+$. An expected change in $[H^+]$ was calculated using the concentrations of **Red** and **Blue** and assuming each **Blue** released 2 protons instead of only 1. This model was close to the experimental data. Exact agreement was not expected because this model does not include any buffering by the TiO_2 NP colloid. A summary of these findings is reported in Table S2.

Table S2 Summary of TiO_2^R (1.2 mM e^-) pH and proton concentration changes before and after oxidation with KI_3.

$pH_{initial}$	pH_{ox}	$[H^+]_{initial}$ (mM)	$[H^+]_{ox}$ (mM)	%**Blue**	%**Red**	Observed $[H^+]$ change (mM)	Expected [a] $[H^+]$ change (mM)
2.68	2.40	2.09	3.98	49.2	50.8	1.89	1.79
2.52	2.29	3.02	5.13	56.6	43.4	2.11	1.88
2.33	2.16	4.68	6.92	65.0	35.0	2.24	1.98
2.24	2.08	5.75	8.32	67.4	32.6	2.56	2.01
2.08	1.96	8.32	10.96	75.4	24.6	2.65	2.10

[a] Calculated as 2 × [**Blue**] + [**Red**], assuming each **Blue** e^- is coupled with 2 H^+ and each **Red** e^- is coupled with 1 H^+.

6. H^+:e^- Stoichiometries for Red and Blue States

Generally, a concentrated stock solution of TiO_2^R (15 mg/mL) was photolyzed in H_2O to generate a substantial concentration of electrons (~10 mM e^-). After the photolysis, the reduced colloids were separated into two batches and were diluted 10-fold into H_2O or D_2O. This resulted in samples that were 100% in H_2O and 90% D_2O, as shown in Scheme S2. Samples were allowed to thermally and chemically equilibrate, and the relative changes of the protic and deuterated samples were evaluated.

The pH (or pH*) of TiO_2^R in H_2O (or TiO_2^R in D_2O) prepared by Scheme S2 were adjusted through the dilution with mixtures of H_2O/HCl (or D_2O/DCl) giving the UV-vis spectra in Figure S8A. This is to maintain a constant volume across samples to ensure identical concentrations of reducing equivalents. In total, 0 to 50.4 μmol of HCl (or DCl) was added to separate solutions of TiO_2^R in H_2O (or TiO_2^R in D_2O). Spectrophotometric titrations of four solutions with 4-MeO-TEMPO showed the colloids all maintained the same electron concentration (0.91 ± 0.04 mM) but had different slopes (Figure S8B).

TiO_2^R in D_2O always had a lower measured pH* than the measured pH for TiO_2^R in H_2O (pH* – pH = –0.3). Although, more variation in pH vs. pH* was observed in the reduced samples than for fully oxidized samples.

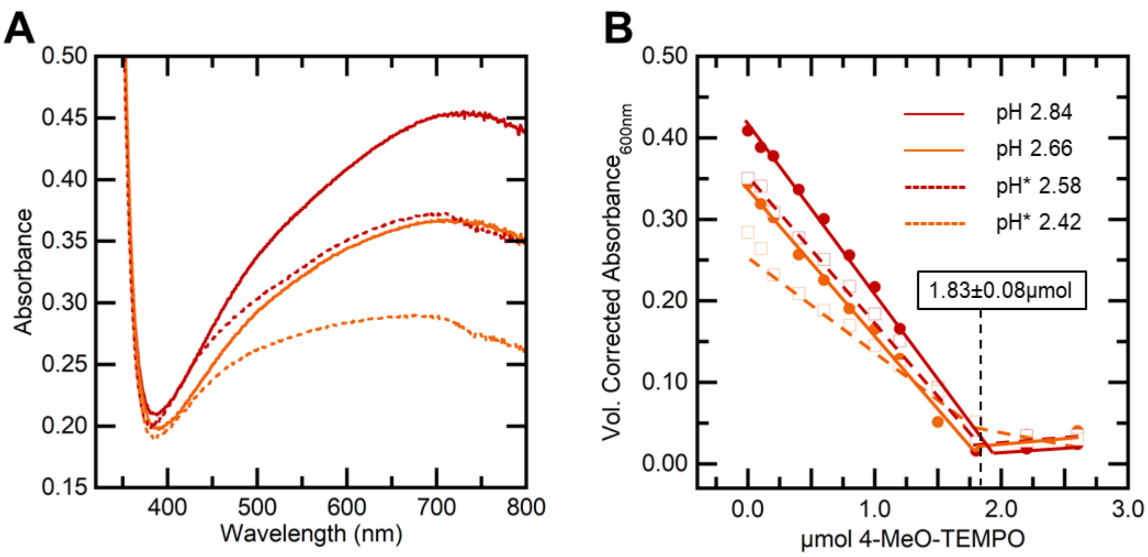

Figure S8. (**A**) UV-vis spectra of TiO_2^R NPs diluted 10-fold into H_2O (red trace, pH 2.84) and D_2O (red dashed trace, pH* 2.58), accounting for the addition of equal amounts of HCl (orange trace, pH 2.66) and DCl (orange dashed trace, pH* 2.42). (**B**) Spectrophotometric titrations of TiO_2^R in H_2O (pH 2.84 and 2.66) and in 90% D_2O (pH* 2.58 and 2.42) with 4-MeO-TEMPO. All samples show a similar endpoint (1.83 ± 0.08 μmol 4-MeO-TEMPO), which suggest the reducing equivalent is unaffected by pH modulation.

7. pH of the TiO_2 NP Colloids

7.1 pH and proton concentrations

The pH measurements determine the proton activity (α_{H^+}), which we assume in this work is equal to proton concentration. The relationship between pH and [H+] is show in equations S1 and S2.

$$pH = -\log_{10}[\alpha_{H^+}] \approx -\log_{10}[H^+] \qquad \text{(eq S1)}$$

$$[H^+] = 10^{-pH} \qquad \text{(eq S2)}$$

7.2 TiO_2 pH in Mixed H_2O and D_2O Solvents

Measurements of D_2O-based solutions using a standard H_2O glass pH electrode, calibrated with standard buffers in H_2O, are denoted pH*. Because the dissociation constant of H_2O is different from D_2O (pK_w^H = 14.00, pK_w^D = 14.96), pH* must be adjusted in heavy water solutions.[10-11] The adjusted value, named pD, is comparable to the pH of analogous H_2O solutions. Traditional methods, typically used closer to neutral pHs, add 0.40 – 0.45 to pH* to obtain pD.[12-15] In this study, we empirically estimated the correction factor to be 0.35 (eq S3), using the procedure below. The D_2O volume fraction is also accounted for.

$$pD = pH^* + (0.35 \times \text{vol. fraction } D_2O) \qquad \text{(eq S3)}$$

The influence of solvent composition on the colloid pH* under these specific conditions was measured as follows. Concentrated TiO_2 NP stock solutions were prepared with either H_2O or D_2O, at 15 mg / mL. A series of

ten 10-fold dilutions was conducted for each sample, resulting in a total of 20 samples in different mixed solvent ratios. In these dilutions, 100 µL of NPs were added into 900 µL of solutions with various D_2O volume fractions ranging from 0 – 1 (Scheme S3). The 20 samples were equilibrated overnight, and their pH* were recorded (Figure S9).

Scheme S3 Experimental design of 10-fold dilutions of TiO_2 in H_2O and D_2O into mixed solvents.

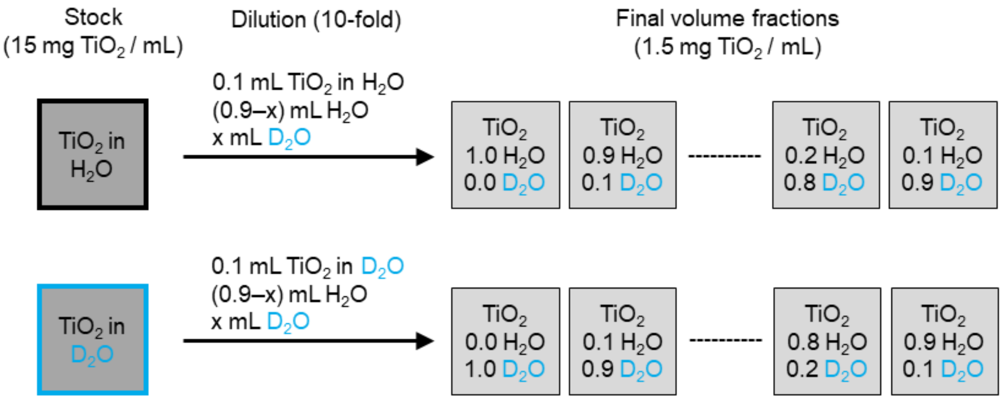

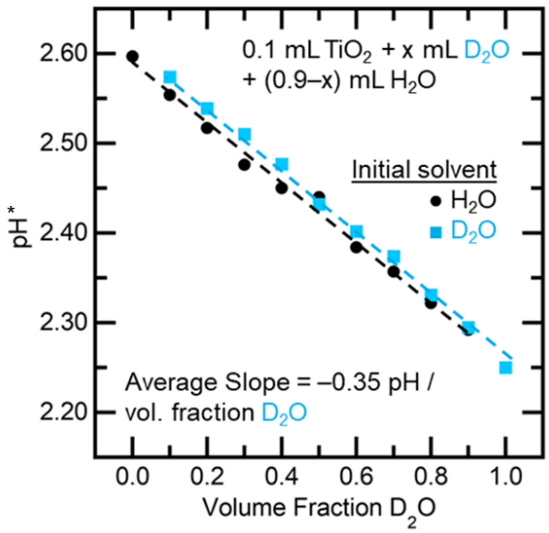

Figure S9. Measured pH* versus volume fraction of D_2O for TiO_2 suspensions. TiO_2 was dissolved in H_2O or D_2O (15 mg / mL) and was diluted 10-fold into samples with various volume fractions of D_2O ranging from 0 – 1. An average linear relationship was determined to be – 0.35 pH units / vol. fraction D_2O.

The measured pH* varied linearly with the final volume fraction of D_2O in the mixed solvent, with an average slope of –0.35 pH units / volume fraction D_2O (Figure S3). There was little difference between samples that originated in H_2O versus D_2O. These measurements are not a rigorous method to determine the relationship between pH and pD (which may not be possible); for instance, the surface groups on the TiO_2 may have different pK_a values in H_2O versus D_2O. Still, the linearity of the plot starting from the same batch of TiO_2 NPs provides some confidence in this estimation.

7.3 Acid titrations in H_2O and D_2O

Acid titrations to oxidized TiO_2 colloids were conducted to further map out the relationship between pH / pH* and [H^+] / [D^+]. Concentrated TiO_2 was prepared in H_2O (15 mg / mL) and was diluted 10-fold into H_2O and D_2O (Scheme 4). Samples were titrated with matched acids (HCl in H_2O, DCl in D_2O, at the same concentrations), and the pH were recorded. As expected, the pH* measured for the samples in D_2O were systematically lower than their respective H_2O/HCl pair upon each acid addition.

Scheme S4 Experimental design for HCl and DCl titrations of TiO_2 colloids presented in Figure .

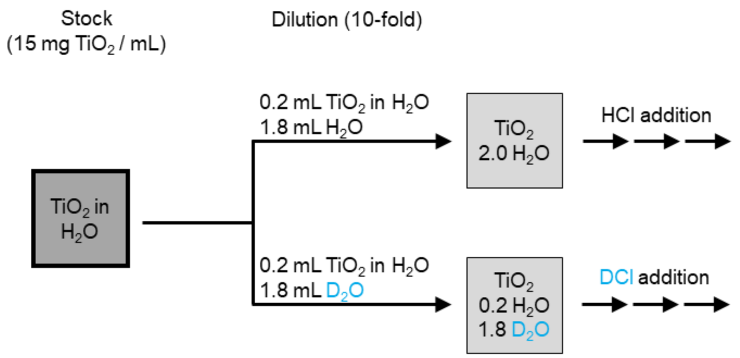

The recorded pH and pH* values were converted into approximate concentrations using eq S2. Concentrations of H^+ and D^+ were calculated assuming no pH* to pD correction was required (without using eq S3). Using this method, Figure S10A shows the D_2O samples had a substantially larger calculated [D^+] than [H^+] even though the same concentrations of HCl and DCl were added to each sample. Thus, it was determined this was not an appropriate method to calculate [D^+].

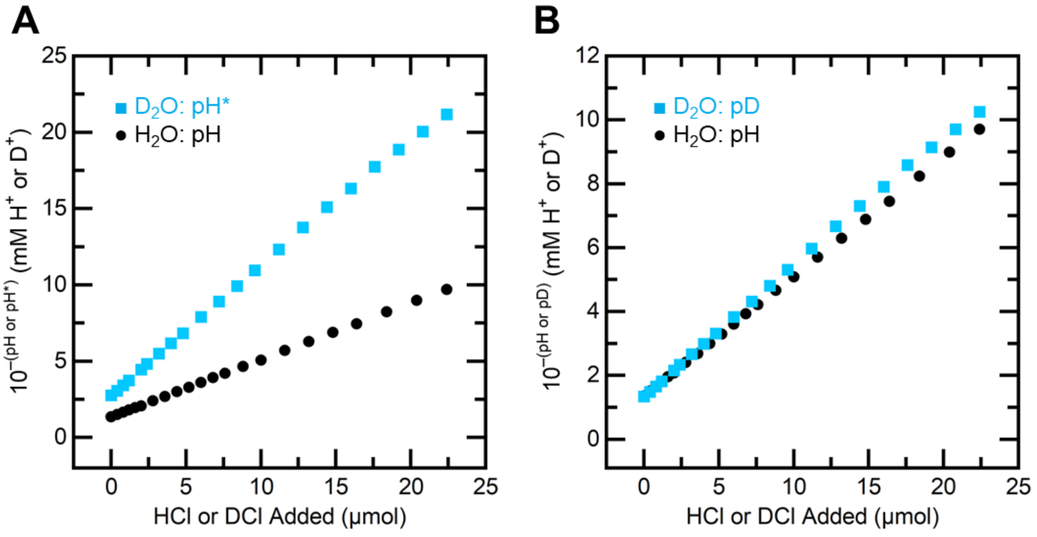

Figure S10. Data from the acid aliquot additions presented in Scheme S4. (**A**) Titration of TiO_2 colloids in 100% H_2O or 90% D_2O with added HCl or DCl and the corresponding pH (or pH*) measured after acid addition. The measured pH for D_2O samples was always systematically lower than H_2O samples without the correction from equation S3. (**B**) The measured pH and corrected pH* (pD) versus added HCl or DCl after correction. The plots show good agreement.

The pH* to pD correction method empirically determined for this system in Figure S9 (eq S3) was applied to the samples from Scheme S4 and Figure S10. As the deuterated samples were 90% D_2O, the expected correction value was ~0.32 to add to each pH* (eq S3). Using this method, the HCl or DCl addition vs pH correlation overlaid almost perfectly (Figure S10B) and was substantially improved from the uncorrected data presented in Figure S10A. To calculate the $[D^+]$, the correction method outlined in eq S3 was applied and can be rearranged into eq S4. Thus, to calculate $[D^+]$ within this system, this method was used.

$$[\mathrm{D}^+] = 10^{-(\mathrm{pH}^*+0.32)} \qquad \text{(eq S4)}$$

8. Trap State Behavior of Citrate-Capped TiO_2 (*c*-TiO_2)

To test the changes in trap state behavior of citrate-capped TiO_2^R (*c*-TiO_2^R) with changes in pH, capped particles were synthesized following the procedure outlined in section S1.2. Briefly, uncapped TiO_2 was added to an aqueous solution of 6 mM citric acid (pH 5.45) and was photolyzed for 4.5 minutes, resulting in a blue colloid (Figure S11A, blue trace). The pH was modified using HCl and TMAOH (see caution above) and spectral changes were observed. Like the uncapped NPs presented in most of these studies, the absorbance increased and redshifted for basic samples (across a much wider range of stable pH in this case), and vice versa for acidic samples. These changes took a significant amount of time to fully equilibrate (up to 5 hours), as monitored optically or by its pH changes. In contrast, the uncapped systems required ~ 5 minutes to equilibrate.

The *c*-TiO_2^R samples above were flash frozen, and EPR spectra were measured (Figure S11B). The *c*-TiO_2^R appeared to have multiple components (perhaps like **Red** and **Blue**). In comparison with the *uc*-TiO_2^R, however, the components of the *c*-TiO_2^R spectra were less well resolved.

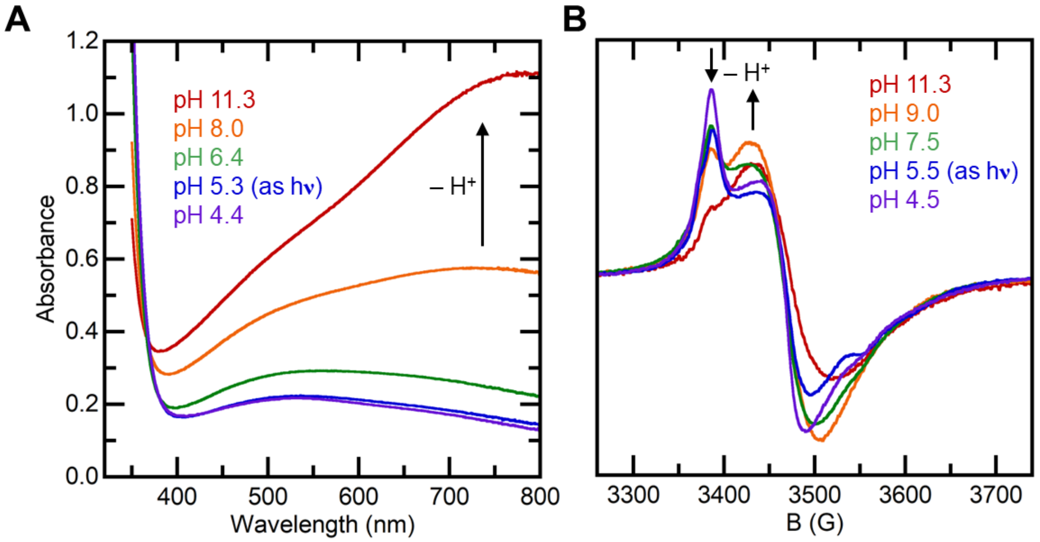

Figure S11. (**A**) UV-vis absorption spectra of *c*-TiO_2^R (6 mM citric acid, pH 5.3) before (labelled "as hν") and after pH modulation using HCl and TMAOH (see *Caution* above). Samples that were more basic increased in absorbance and the absorbance maximum redshifted. These changes were similar to those observed for *uc*-TiO_2^R, though the *c*-TiO_2^R required ~5 hours to equilibrate. (**B**) EPR spectra of *c*-TiO_2^R (6 mM citric acid, pH 5.3) post photolysis and after equilibration with added TMAOH (pH 11.3, 9.0, 7.5) or HCl (pH 4.5).

Nevertheless, the "as-photolyzed" *c*-TiO_2^R sample (pH 5.5) was compared to basified (pH 11.3, 9.0, and 7.5) samples and acidified (pH 4.5) samples by EPR (Figure S11B). These samples followed a similar pattern to *uc*-TiO_2^R, where the lowest magnetic field feature of the EPR spectrum decreased as the pH increased (similar to **Blue** decreasing at higher pH). Qualitatively, this matches the trends observed with uncapped particles.

9. Effect of Formic Acid/Formate on Trap State Proton Equlibrium of TiO_2^R

Upon photoreduction of TiO_2 to TiO_2^R using CH_3OH (150 mM) as a sacrificial reductant, formic acid (δ 8.2 ppm) can be detected via ^1H NMR spectroscopy (Figure S12).

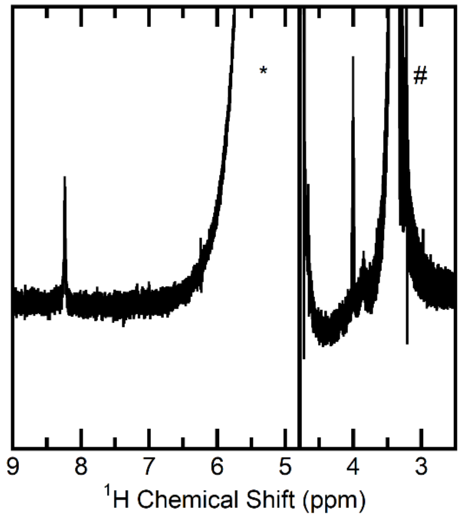

Figure S12. ^1H NMR spectrum of photolyzed TiO_2 in the presence of 150 mM CH_3OH. * Indicates H_2O, and # indicates CH_3OH. Formic acid (δ 8.2 ppm) can be observed.

We then evaluated the effect (or lack thereof) of formic acid and formate anion on the TiO_2^R trap state equilibrium. Photolysis of TiO_2 (3 mg/mL) yielded TiO_2^R at pH 2.91 (black trace in Figure S13A, and black dot in Figures S13B,C). Additions of HCOOH (0.4 μmol aliquots from a 20 mM stock solution) resulted in the red (0.4 μmol total), orange (0.8 μmol), green (1.2 μmol), and purple (2.0 μmol) optical spectra in Figure S13A, which showed little changes. Concurrent pH measurements (circles in Figure S13B) also showed a lack of change in the solution pH. Overall, we conclude formic acid does not affect the trap state equilibrium. Afterwards, an addition of potassium formate (2.0 μmol from a 20 mM stock solution) yielded the purple optical spectrum (Figure S13A) and resulted in an increase in pH (purple circle in Figure S13C). These observations can be explained by formate's role as a base (the pK_a of formic acid is 3.75), and its addition increased the solution pH and therefore shifted the trap state equilibrium towards **Red**.

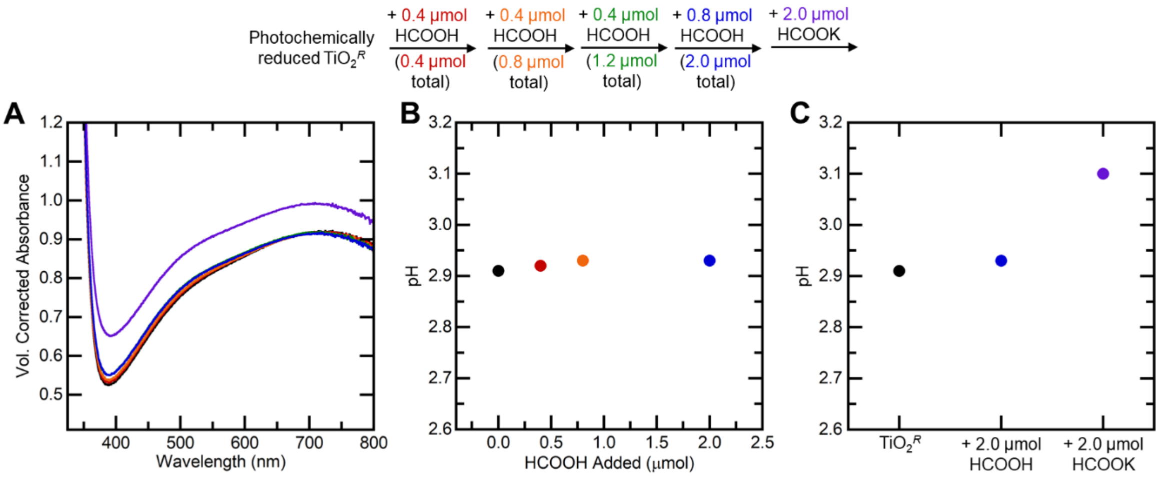

Figure S13. (**A**) Optical spectra of TiO_2^R before (black) and after the additions of 0.4 μmol (red), 0.8 μmol (orange), 1.2 μmol (green), 2.0 μmol (blue) HCOOH, and 2.0 μmol HCOOK (purple). (**B**) pH measurements of the TiO_2^R colloidal solution before (black circle) and after the additions of 0.4 μmol (red), 0.8 μmol (orange), and 2.0 μmol (blue) HCOOH. (**C**) pH measurements of the TiO_2^R colloidal solution before (black circle) and after the additions of 2.0 μmol HCOOH (blue), and 2.0 μmol HCOOK (purple).

10. References for Supporting Information

(1) Peper, J. L.; Gentry, N. E.; Boudy, B.; Mayer, J. M., Aqueous TiO_2 Nanoparticles React by Proton-Coupled Electron Transfer. *Inorganic Chemistry* **2022,** *61* (2), 767-777. https://doi.org/10.1021/acs.inorgchem.1c03125

(2) Peper, J. L.; Vinyard, D. J.; Brudvig, G. W.; Mayer, J. M., Slow Equilibration between Spectroscopically Distinct Trap States in Reduced TiO_2 Nanoparticles. *Journal of the American Chemical Society* **2017,** *139*, 2868-2871. https://doi.org/10.1021/jacs.6b12112

(3) Mohamed, H. H.; Mendive, C. B.; Dillert, R.; Bahnemann, D. W., Kinetic and Mechanistic Investigations of Multielectron Transfer Reactions Induced by Stored Electrons in TiO_2 Nanoparticles: A Stopped Flow Study. *Journal of Physical Chemistry A* **2011,** *115*, 2139-2147. https://pubs.acs.org/doi/pdf/10.1021/jp108958w

(4) Peper, J. L.; Gentry, N. E.; Brezny, A. C.; Field, M. J.; Green, M. T.; Mayer, J. M., Different Kinetic Reactivities of Electrons in Distinct TiO_2 Nanoparticle Trap States. *Journal of Physical Chemistry C* **2021,** *125* (1), 680-690. https://doi.org/10.1021/acs.jpcc.0c10633

(5) Panayotov, D. A.; Burrows, S. P.; Morris, J. R., Photooxidation Mechanism of Methanol on Rutile TiO_2 Nanoparticles. *Journal of Physical Chemistry C* **2012,** *116* (11), 6623-6635. https://pubs.acs.org/doi/pdf/10.1021/jp209215c

(6) Augugliaro, V.; Bellardita, M.; Loddo, V.; Palmisano, G.; Palmisano, L.; Yurdakal, S., Overview on Oxidation Mechanisms of Organic Compounds by TiO_2 in Heterogeneous Photocatalysis. *Journal of Photochemistry and Photobiology C: Photochemistry Reviews* **2012,** *13* (3), 224-245. https://www.sciencedirect.com/science/article/pii/S138955671200024X?via%3Dihub

(7) Seraghni, N.; Belattar, S.; Mameri, Y.; Debbache, N.; Sehili, T., Fe(III)-Citrate-Complex-Induced Photooxidation of 3-Methylphenol in Aqueous Solution. *International Journal of Photoenergy* **2012,** *2012*, e630425. https://downloads.hindawi.com/journals/ijp/2012/630425.pdf

(8) Mudunkotuwa, I. A.; Grassian, V. H., Citric Acid Adsorption on TiO_2 Nanoparticles in Aqueous Suspensions at Acidic and Circumneutral pH: Surface Coverage, Surface Speciation, and Its Impact on Nanoparticle−Nanoparticle Interactions. *Journal of the American Chemical Society* **2010,** *132* (42), 14986-14994.

(9) Stoll, S.; Schweiger, A., EasySpin, A Comprehensive Software Package for Spectral Simulation and Analysis in EPR. *Journal of Magnetic Resonance* **2006,** *178*, 42-55. https://www.sciencedirect.com/science/article/pii/S1090780705002892?via%3Dihub

(10) Harned, H. S.; Robinson, R. A., A note on the temperature variation of the ionisation constants of weak electrolytes. *Transactions of the Faraday Society* **1940,** *36* (0), 973-978. http://dx.doi.org/10.1039/TF9403600973

(11) Covington, A. K.; Robinson, R. A.; Bates, R. G., The Ionization Constant of Deuterium Oxide from 5 to 50°. *The Journal of Physical Chemistry* **1966,** *70* (12), 3820-3824. https://doi.org/10.1021/j100884a011

(12) Krężel, A.; Bal, W., A formula for correlating pK_a values determined in D_2O and H_2O. *Journal of Inorganic Biochemistry* **2004,** *98* (1), 161-166. https://doi.org/10.1016/j.jinorgbio.2003.10.001

(13) Covington, A. K.; Paabo, M.; Robinson, R. A.; Bates, R. G., Use of the glass electrode in deuterium oxide and the relation between the standardized pD (paD) scale and the operational pH in heavy water. *Analytical Chemistry* **1968,** *40* (4), 700-706. https://doi.org/10.1021/ac60260a013

(14) Glasoe, P. K.; Long, F. A., Use of Glass Electrodes to Measure Acidities in Deuterium Oxide. *Journal of Physical Chemistry* **1960,** *64* (1), 188-190. https://doi.org/10.1021/j100830a521

(15) Mikkelsen, K.; Nielsen, S. O., Acidity Measurements with the Glass Electrode in H_2O-D_2O Mixtures. *Journal of Physical Chemistry* **1960,** *64* (5), 632-637. https://doi.org/10.1021/j100834a026
