# Supplementary material for: Trap States in Reduced Colloidal Titanium Dioxide Nanoparticles Have Different Proton Stoichiometries
Source: ACS Cent Sci. 2024 Nov 22;10(12):2266–73. doi: 10.1021/acscentsci.4c01074 (PMC11672544; doi:10.1021/acscentsci.4c01074)
Supplement: Supplementary file 2 — oc4c01074_si_002.pdf [file oc4c01074_si_002.pdf]

oc-2024-01074y.R1

Name: Peer Review Information for "Trap States in Reduced Colloidal Titanium Dioxide Nanoparticles Have Different Proton Stoichiometries"

First Round of Reviewer Comments

Reviewer: 1

Comments to the Author

The manuscript by Gentry et al. describes some intriguing observations related to proton-coupled electron transfer in TiO2 nanoparticles. Specifically they identify 2 electron trap states binding either one or two protons upon trap filling. The experiments are very well performed, but I have some doubts the general aspects of these observation and about their origin:

1. TiO2 np are reduced upon UV irradiation in an aqueous solution containing methanol during several hours. Are the TiO2 np unchanged after this procedure (besides the fact that they are reduced)? Methanol oxidation could lead to formation of several intermediates such as formic acid that could bind to the TiO2 surface and potentially affect the following experiments. FTIR might be a useful tool.

2. Can the reduction of TIO2 np be achieved in a different way to avoid methanol? Are similar results obtained for larger more crystalline TIO2 pn?

3. Upon reduction, the spectra of TiO2 np show a broad absorption feature in the visible range. In the UV range, there does not seem to any change. In spectroelectrochemical measurements of mesoporous TiO2 electrodes, however, a bleach is observed, due to a decrease in TiO2 conduction band states (or CB filling). Why this difference?

Reviewer: 2

Comments to the Author

The authors follow up previous work in JACS and provide compelling evidence for two types of reduced trap states in aqueous colloidal TiO2 at 10 K. Titration and deuterium isotope studies indicate that one state has a single proton and the other two and that these states are in

equilibrium. This is carefully done work and the conclusions are mainly supported by the experimental data.

I am however very lukewarm about publication of this work in a high impact journal and believe it is better suited for a more specialized journal. Only one type of apparently amorphous TiO2 (there is more interest in the rutile and anatase polymorphs) and the generality is hence suspect.

In addition, the authors appear to make no distinction between aqueous colloidal nanoparticles and the wide variety of technologically important semiconductor interfaces when they make assertions like: "ubiquitous assumption that interfacial trap states are purely electronic" that is repeated in various guises throughout the text. This reviewer simply does not believe that this is an accurate description of the field and would be confusing to broad readership. At colloidal TiO2 aqueous interfaces the dogma is that the trap states energies change with pH as do the band edges. In mesoporous anatase TiO2 thin films, Fitzmaurice and coworkers monitored a very similar absorption and concluded that it shifted 59 mV/pH unit. They did assign the coloration to CB electrons but whether these were trapped or free electrons at RT remains unclear in this prior work and in this submission. Goosens and Boschloo also provided evidence for two types of trap states in these arguably more relevant materials. In the vast TiO2 aqueous colloid literature there may be a papers where electronic strap states are invoked that are pH independent (often because they had no way to probe this), but the dogma in the field is that the trap states and the band edges all move together with pH.

In support of this assertion, references 7, 13-17 are listed. This too seems misleading. Reference 7 describes new methods for the characterization of charge trapping many of which were not performed at aqueous interfaces at all. The same is true for Bisquert's work that was mainly developed for DSSCs based on TiO2 at non-aqueous interfaces where Li+ cations play a more important role. The vast majority of SC interfaces, and arguably the most important technologically, are not with aqueous solutions and this important point is never made.

Some additional points to consider:

The concentration of electrons in TiO2 as mM was confusing. Is this in the solution or in the TiO2? How many electrons on average were present in each nanoparticle?

The EPR data was analyzed carefully, but not the UV-Visible spectra. What absorption spectrum of the blue and red would enable modelling of their combined spectra? Are the equilibrium constants in agreement with the EPR data?

Is it fair to compare the 10K epr data with the room temperature data? Presumably the equilibrium constant is temperature dependent.

The change in the proton concentration for the blue was 50-100% larger than for the red; doesn't this imply that the stoichiometry for the blue is less than 2? Random error would give values > 100% if it was exactly 2. How does one rule out a distribution of proton stoichiometries for the blue?

The physical location of the trap states may not be accessible surface sites, as was suggested, especially if the anatase polymorph is present that has internal channels that can host protons

Reviewer: 3

Comments to the Author

This study demonstrates that the different traps in TiO_2 nanocrystals (previously deemed "red" and "blue" by the same group) have different proton stoichiometries. Specifically, it is shown that the two traps are in protic equilibrium, where adding a proton to the red trap yields a blue trap, which behaves analogously to a soluble molecular acid. This work highlights the importance of considering chemical stoichiometries in addition to electronic/structural properties when describing traps in semiconductor materials and provides a platform for understanding reactivity at metal oxide surfaces. It is thus of interest to the broad semiconductor and nanocrystal communities and is recommended for publication in ACS Central Science. One minor revision is suggested below.

It is very clearly demonstrated that the red trap has one less proton than the blue trap. The 2:1 ratio, however, was not quite as clear. It seems like this was claimed based on the data presented in Figure 3. It is stated that "the model of eqs 5 and 6 most closely matches behavior of the experimental data." However, the experimental data (blue circles) only matches this model (black dashed line) at one data point (~50% blue). If the trend of the experimental data is extended to lower % blue, it would cross the model for 1 H^+ on blue and 0H^+ on red (purple dashed line). I understand that a perfect agreement is not expected due to buffering from the nanocrystals, but

can the authors elaborate on how it was determined that the better match was to the 2:1 model? Perhaps collecting a couple data points at lower %blue would make this more clear.

Author's Response to Peer Review Comments:

Yale University

James M. Mayer
Charlotte Fitch Roberts Professor of Chemistry
Department of Chemistry
225 Prospect Street / P.O. Box 208107
New Haven, CT 06520-8107
james.mayer@yale.edu, (203) 436-9456

September 12, 2024

Deputy Editor, *ACS Central Science*
RE: Manuscript ID: oc-2024-01074y

Title:
"Trap States in Reduced Colloidal Titanium Dioxide Nanoparticles Have Different Proton Stoichiometries"
Authors: Noreen Gentry, Noah J. Gibson, Justin L. Lee, Jennifer L. Peper, James M. Mayer

Dear Editor,

Thank you for sending the reviews of the manuscript revision referenced above. We are pleased that all of the reviews ranked the technical rigor as Top 5%, and that two of the three ranked the significance, broad interest, and novelty in the Top 5% (or better).

We have revised the manuscript to address the concerns raised by reviewers. In the following pages of this letter, the reviews have been reproduced in Arial font. Below each point, in blue Times New Roman font, we describe how the manuscript has been revised to address that concern. Text quoted from the revised manuscript is indented and in burgundy text. A copy of the manuscript with the important changes shown in orange has been uploaded as Supporting Information for Review Only, along with the updated Supporting Information.

Because a new set of experiments has been added (Section 1A), a new author has been added, Jennifer L. Peper.

We hope that the revised manuscript will prove acceptable for publication.

Thank you again for your attention to this paper.

Sincerely,

James M. Mayer
Charlotte Fitch Roberts Professor of Chemistry

Reviewer 1

Recommendation: Reconsider after major revisions noted.

Additional Questions:
Quality of experimental data, technical rigor: Top 5%
Significance to chemistry researchers in this and related fields: High
Broad interest to other researchers: Top 5%
Novelty: Top 5%
Is this research study suitable for media coverage or a First Reactions (a News & Views piece in the journal)?: Yes

Comments:

The manuscript by Gentry et al. describes some intriguing observations related to proton-coupled electron transfer in TiO2 nanoparticles. Specifically, they identify 2 electron trap states binding either one or two protons upon trap filling. The experiments are very well performed, but I have some doubts the general aspects of these observation and about their origin:

We thank the reviewer for all of their valuable comments, which have improved the manuscript.

TiO2 np are reduced upon UV irradiation in an aqueous solution containing methanol during several hours. Are the TiO2 np unchanged after this procedure (besides the fact that they are reduced)?

Spectroscopic data indicate that the TiO_2 NPs do not undergo major changes upon oxidation and reduction. The optical spectra of the as-prepared nanoparticles, and that after a reduction-then-oxidation cycle are the same, with no shift of the band edge adsorption or broadening of the band edge that might indicate agglomeration.[1-2] [references are given at the end of this letter] EPR spectra are essentially the same in the first reduction and after a reduction/oxidation/reduction cycle. TEM and PXRD are problematic in this system because of their coagulation under high vacuum (below 20 torr in our experience) and the high air sensitivity of the reduced NPs.

We have been working with this colloid system for a decade and we have no evidence for significant changes upon reduction. Reversible redox equilibria were demonstrated with a variety of redox reagents.[2] Time-resolved partial oxidations of the reduced TiO_2 colloids consume mostly the 'Red' electrons, and then re-equilibration restores the same equilibrium ratio observed upon photoreduction.[3] We expect that some titanium ions likely dissolve in the solution, since TiO_2 has a small solubility in water below pH 3 and Ti^{3+} complexes are more soluble. But whatever changes may be occurring do not appear to affect the Red/Blue/oxidized stoichiometry and equilibria (see also the Cr(II) chemistry added to the manuscript, as described below).

Methanol oxidation could lead to formation of several intermediates such as formic acid that could bind to the TiO2 surface and potentially affect the following experiments. FTIR might be a useful tool.

The reviewer is correct that formic acid is formed by photolysis: a small amount is observed in ^{1}H NMR spectra of the TiO_2 colloids after photolysis (see new SI section 9). Unfortunately, FTIR spectra are problematic for these materials because the isolated NP powder is only partially dried and contains substantial water and HCl, so the carboxylate region of their spectra is obscured. (If the TiO_2 NPs are taken closer to dryness, they change and do not resuspend)

The reviewer asked about formate being involved in the trap state equilibrium. To test this, formic acid was added to reduced TiO_2 suspensions. No shift in the optical spectra were observed up to 1 mM formic acid (approximately the typical $[e^-]$ in photo-reduced $TiO_2{}^R$ NP colloids). The addition of potassium formate caused an upward shift in the spectra, but this shift was expected from the addition of a base and a rise in the pH (the suspensions were at pH 2.91, below the pK_a of formic

acid (3.75); the pH after formate addition was 3.10). These new experiments (SI section 9) rule out the involvement of formic acid/formate in the trap state equilibrium.

Can the reduction of TIO2 np be achieved in a different way to avoid methanol?

Yes, this is an excellent point. Experiments have been added in which the TiO_2 NP colloids were reduced by aqueous Cr^{2+}, rather than by photolysis. This Cr^{2+} chemistry was reported in a thesis[4], and is described in a manuscript to be submitted (a draft is uploaded as Supporting Information for review only[5]). These experiments are described in a new Section 1A (copied below) and its accompanying Figure 3.

1A. Chemically reduced TiO_2^R

Chemical reduction of TiO_2 was also explored to determine whether the **Blue**/**Red** equilibrium described above is related to the photo-production of TiO_2^R. We had previously observed that aqueous Cr^{2+} formed an equilibrium with these colloidal TiO_2, showing the same broad optical spectrum as for photo-reduced TiO_2^R (eq 3, Fig. 3).[4,5]

$$TiO_2 + nCr^{2+} + mH^+ \rightleftharpoons TiO_2^R(\bullet ne^-, mH^+) + nCr^{3+} \qquad (3)$$

Changing the pH of this equilibrium mixture with HCl and TMAOH has two effects (Fig. 3). Addition of acid favors TiO_2^R because reduction of TiO_2 is proton-coupled, shifting eq 3 toward the right. Addition of base reverses the changes. The more subtle effect of acidification is the change in shape of the TiO_2^R spectrum: the absorbance at 800 nm drops more than that at 600 nm. This is the same trend as was observed for photo-reduced TiO_2^R (Fig. 1). These data are more difficult to analyze quantitatively because of the two effects and the absorbance from chromium product(s). Still, the observation of the same reversible spectral response shows that the **Blue**/**Red** equilibrium in eqs 1 and 2 is independent of the method of formation of TiO_2^R.

Are similar results obtained for larger more crystalline TIO2 NP?

In our experience, these TiO_2 NPs are unique in having two and only two clearly distinguishable classes of trap states. In other NPs (such as the same NPs capped with citrate ligands[2]), in our thin films,[6] and in the extensive TiO_2 literature, there are typically many types of trap states (typically observed by EPR).[7-8] The kinds of equilibrium studies reported here are simply not possible for the more typical samples of TiO_2: With many classes of trap states, the broad optical and EPR spectra are not easily deconvoluted. Our belief is that this is a very general phenomenon and that we were lucky and aware enough to have a system where it can be studied. The revised manuscript now makes this point in a new paragraph in the Conclusions.

Upon reduction, the spectra of TiO2 np show a broad absorption feature in the visible range. In the UV range, there does not seem to any change. In spectroelectrochemical measurements of mesoporous TiO2 electrodes, however, a bleach is observed, due to a decrease in TiO2 conduction band states (or CB filling). Why this difference?

We do not know the answer to this question. There have been many studies of TiO_2 thin films, from dense calcined ones to nanoparticulate films, and we are not sure which the reviewer is referring to.[9] To our knowledge, those studied by electrochemistry all show a pH-dependent reduction (Hupp and Lyons's study over 26 orders of magnitude in proton activity). To our knowledge, there has been no systematic study of band edge shifts or their absence. Perhaps the cases that the reviewer is familiar with are much more highly reduced than the particles studied here (only ~3-5% reduced), which would account for a much more pronounced Burnstein-Moss shift for the films.[10]

Reviewer 2

Recommendation: Reconsider after major revisions noted.

Additional Questions:

Quality of experimental data, technical rigor: Top 5%
Significance to chemistry researchers in this and related fields: Moderate
Broad interest to other researchers: Moderate
Novelty: Moderate
Is this research study suitable for media coverage or a First Reactions (a News & Views piece in the journal)?: No

Comments

The authors follow up previous work in JACS and provide compelling evidence for two types of reduced trap states in aqueous colloidal TiO2 at 10 K. Titration and deuterium isotope studies indicate that one state has a single proton and the other two and that these states are in equilibrium. This is carefully done work and the conclusions are mainly supported by the experimental data.

We thank the reviewer for their positive comments.

I am however very lukewarm about publication of this work in a high impact journal and believe it is better suited for a more specialized journal. Only one type of apparently amorphous TiO_2 (there is more interest in the rutile and anatase polymorphs) and the generality is hence suspect.

Text has been added the beginning of the Results to provide more characterization of the NPs (below). Our NPs are primarily anatase, following the colloid preparation and analysis reported by Bahnemann and co-workers in 2011 (that we have repeated in our prior papers on this system). References are given in the text of the paper.

> As in prior studies, the TiO_2 colloids were prepared by hydrolysis of $TiCl_4$ in 16 MΩ water. The particles are mostly anatase, with an average diameter (TEM) of ~4 nm; 1000 Ti atoms / NP based on previous studies. The colloids had [Ti]total = 28 mM (by ICP-MS), corresponding to 30 μM NPs, and they were pH ~ 2.3 after dialysis.

We agree with the reviewer that more systems would provide more evidence of generality. However, the types of studies here do not appear to be possible for other TiO_2 samples, as these usually have much more complex patterns of trap states. This is described in a response to reviewer 1 above, and is now as explained in a new paragraph in the Conclusions Section:

> This study was possible because these TiO_2 NPs have two and only two distinguishable classes of trap states. Typically, oxide semiconductors have many types of trap states, preventing quantitative analysis. We believe that many other systems likely have trap states that change their composition upon population, but to our knowledge this is the first report of such behavior.

For example, these TiO_2 NPs with added citrate have n-type trap states that seems to fall in multiple classes and are not amenable to deconvolution.[2] Other representative systems can be found in references [7-8]. The complexity of most trap state systems is likely a reason why pH-dependent equilibria between trap states have not been previously reported.

The best evidence for the generality of proton-coupled trap state equilibrium is the common super-Nernstian behavior of metal oxide nanoparticles and films (>59 mV/pH; almost as common as Nernstian behavior, particularly for hydrous materials[11-13]). The observation of $2H^+/e^-$ for a discrete trap state is a rare *explanation* of super-Nernstian behavior. We are *proposing* in this report that non-$1H^+/1e^-$ stoichiometries for oxide trap states is likely general. Perhaps we could gently turn the tables on the reviewer's concern: what evidence is that that trap states of different stoichiometries are not common at solid/solution interfaces?

In addition, the authors appear to make no distinction between aqueous colloidal nanoparticles and the wide variety of technologically important semiconductor interfaces when they make assertions like: "ubiquitous assumption that interfacial trap states are purely electronic" that is repeated in various guises throughout the text. This reviewer simply does not believe that this is an accurate description of the field and would be confusing to broad readership. At colloidal TiO2 aqueous interfaces the dogma is that the trap states energies change with pH as do the band edges. In mesoporous anatase TiO2 thin films, Fitzmaurice and coworkers monitored a very similar absorption and concluded that it shifted 59 mV/pH unit. They did assign the coloration to CB electrons but whether these were trapped or free electrons at RT remains unclear in this prior work and in this submission. Goosens and Boschloo also provided evidence for two types of trap states in these arguably more relevant materials. In the vast TiO2 aqueous colloid literature there may be a papers where electronic strap states are invoked that are pH independent (often because they had no way to probe this), but the dogma in the field is that the trap states and the band edges all move together with pH.

We apologize for our misleading text. The revision provides a clarification of the confusing "purely electronic" text, and the tone has been softened. The sentence in the Abstract that the reviewer objected to has been revised (copied below). We have replaced to "ubiquitous" with "common" and sharpened the focus on the chemical change that occurs in the trap states when their populations change.

> Because the trap states have different compositions, their population and depopulation occur with the making and breaking of chemical bonds, not (as commonly assumed) just by the movement of electrons.

The assumption we referred to is that the energies of trap or defect states are discussed as electronic energies, with no change in atomic stoichiometry when the trap state receives or loses an electron. Following the reviewer's use of a DSSC example (in the next point), consider the trapping of a photo-injected e^- in the semiconductor. We are not aware of any papers that propose that this trapping step involves a change in the chemical stoichiometry of the 'trap state,' other than the addition or removal of an electron.

We completely agree with the reviewer that the pH dependence of band energies has long been known, for at least a half century. *Yes*, Fitzmaurice, Goosens, Boschloo, Grätzel, and many others demonstrated Nernstian behavior for TiO_2 decades ago. *Yes*, Hupp and Lyons in 1999 showed Nernstian behavior for their TiO_2 films over 26 orders of magnitude in proton activity.[14] Based on that work and many other studies, we argued in a recent *JACS* Perspective that such PCET reactions are ubiquitous at solid-solution interfaces when there are basic or protic sites at the solid surface and an even small proton activity in solution.[15] Morrison's classic books from 1980 and 1990 derive this behavior for semiconductor interfaces.

> S. R. Morrison, *Electrochemistry at semiconductor and oxidized metal electrodes*, Plenum Press 1980, especially Chapter 2: The Solid/Liquid Interface, especially Section 2.2.3, and Chapter 5: The Properties of the Electrode and Their Effect on Electrochemical Measurements, Section 5.1.

> S. R. Morrison, Chapter 8 *The Solid/Liquid Interface*, in *The Chemical Physics of Surfaces*, edited by S. R. Morrison, Springer US 1990, Section 8.2, esp. pages 267-276.

However, Morrison's books derive Nernstian behavior for semiconductor interfaces as due to charging of the surface by protonation. This requires the assumption that the surface coverage of H^+ changes *linearly* with the solution proton activity. For instance, (text from the 1990 book cited above; italics added):

> the relationship for insulators between the Helmholtz double-layer potential V_H and the surface charge $[H_s^+]'$ where the latter symbol represents the net density of adsorbed protons. In the reaction, ….

$$H_3O^+ = H_s^+ + H_2O \qquad (8.1)$$

$$eV_H = B + kT\ln[H_3O^+] = B - 2.3kT\,(pH) \qquad (8.4)$$

the Helmholtz double layer is insensitive to electron transfer …. the net current density … becomes

$$J = \sigma\bar{c}e\, d(kT/\pi\lambda)^{1/2} c_{ox} \exp[-(E_{cs} - E_{ox})^2/4\lambda kT](n_s - n_{s0}) \qquad (8.15)$$

…. [where] n_s [is] the density of *current carriers at the semiconductor surface* [i.e., electrons or holes]. …. recalling that the only variable in (8.15) with voltage is n_s, we can write Equation (8.15) in terms of an exchange current J_o:

$$J = J_0(1 - n_s/n_{s0}) \qquad (8.20)$$

There is no connection to trap/defect states in this model, and the energies of the states within semiconductor clearly refer to electronic energies, not free energies that would include addition or removal of protons. The papers referred to in the review do not assign the protons to trap states, the protons simply bind to the surface. Those papers do not suggest that when an e^- is added to or removed from a trap state, there is a concomitant transfer of a fixed number of protons per electron. We believe that our revised text makes these points clear to readers.

In support of this assertion, references 7, 13-17 are listed. This too seems misleading. Reference 7 describes new methods for the characterization of charge trapping many of which were not performed at aqueous interfaces at all. The same is true for Bisquert's work that was mainly developed for DSSCs based on TiO2 at non-aqueous interfaces where Li+ cations play a more important role. The vast majority of SC interfaces, and arguably the most important technologically, are not with aqueous solutions and this important point is never made.

We respectfully disagree that "the vast majority of SC interfaces, and arguably the most important technologically, are not with aqueous solutions." The classic Fujishima and Honda paper on water splitting at a TiO_2 electrode (1972) was done in water. The book *Semiconductor Electrodes* edited by H. O. Finklea (Elsevier 1988) is dominated by aqueous electrochemistry (as are Morrison's books cited above). Band diagrams referenced to *aqueous RHE* are ubiquitous in the literature for essentially all semiconductors. A few classic band diagrams from giants in the field (Nozik, Grätzel, Domen) are copied below. These show many of the major semiconductors, and their energies *in water* (often specifying the aqueous pH and setting the O_2/H_2O couple at the aqueous 1.23 V). A major reason that TiO_2 and other semiconducting oxides like NiO are being widely used is because of their stability in water, at least compared with Si. The large literature of the electrochemistry of silicon—the most technologically important semiconductor—is predominantly in aqueous solutions, not non-aqueous ones. The $60M DOE-funded LISA Solar fuels Hub is focused on metal catalysts supported on semiconductors mostly in water (as was the prior "Powering the Planet" center). Commercial photoelectrochemical processes at energy scale, such as water splitting or CO_2 reduction, will likely be done in water to avoid the higher resistive losses and cost of organic solvents. Plus, water will be a reagent (water splitting) or a product (CO_2 reduction).

We also defend the citing of papers both from the aqueous and non-aqueous literatures. We believe that these principles will prove valuable to both. Our laboratory has shown that the electrochemistry of NiO films is *the same* in both water and non-aqueous buffered (e.g., pyridine/pyridinium) electrolytes[16], and there are strong similarities for TiO_2 as well.[2,6]

Photoelectrochemistry: Applications to Solar Energy Conversion, A. J. Nozik, *Annual Review Of Physical Chemistry* **1978**, 29, 189-222.
Photoelectrochemical cells M. Grätzel *Nature* **2001**, *414* 338 – 344.
Electrochemical Photolysis of Water at a Semiconductor Electrode A. Fujishima and K. Honda *Nature* **1972**, *238*, 37-38.
Particulate photocatalysts for overall water splitting Shanshan Chen, Tsuyoshi Takata & Kazunari Domen *Nature Reviews Materials* **2017**, *2*, 17050.

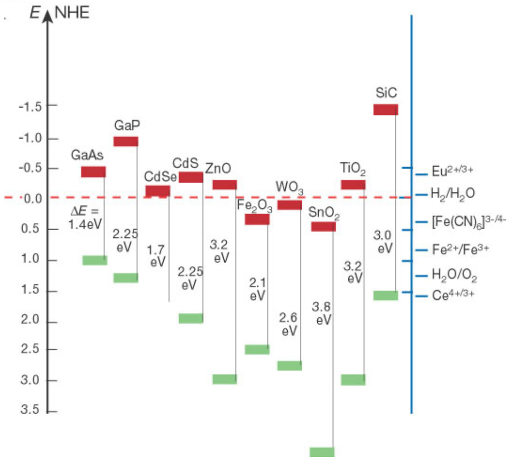

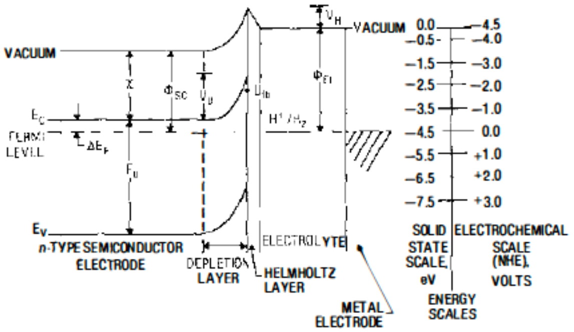

Figure 1 Energy level diagram for semiconductor-electrolyte junction showing the relationships between the electrolyte redox couple (H^+/H_2), the Helmholtz layer potential drop (V_H), and the semiconductor band gap (E_g), electron affinity (χ), work function (ϕ_{sc}), band bending (V_B), and flat-band potential (U_{fb}). The electrochemical and solid state energy scales are shown for comparison. ϕ_{El} is the electrolyte work function.

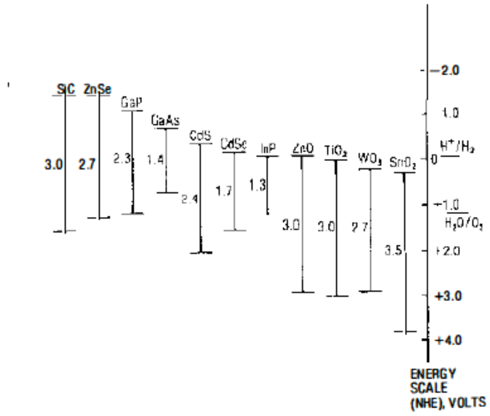

Figure 2 Position of valence and conduction band edges for several semiconductors in contact with aqueous electrolyte at pH = 1.0. The position of the H^+/H_2 and H_2O/O_2 redox couples are indicated at the right.

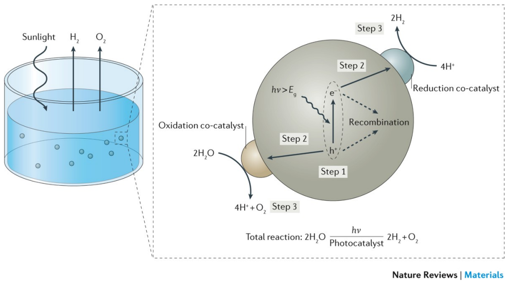

The concentration of electrons in TiO2 as mM was confusing. Is this in the solution or in the TiO2? How many electrons on average were present in each nanoparticle?

These questions have been answered (and clarified) in the revised the first paragraph of the Results in Discussion. The concentration of electrons is the moles of electrons per liter of colloid solution (12 mM e^- for the undiluted sample), as that is what is determined by solution titration. The number of electrons per TiO_2 NP is ~40 e^-/NP (12 mM e^- / 150 μM NP before dilution) used in most of the experiments. The number of electrons per NP does not change with dilution.

The EPR data was analyzed carefully, but not the UV-Visible spectra. What absorption spectrum of the blue and red would enable modelling of their combined spectra? Are the equilibrium constants in agreement with the EPR data?

This has been discussed in our prior work with these NPs.[1] The optical spectra are broad, overlapping and featureless, and do not uniquely separate into specific "red" and "blue" spectra. The EPR spectra are much more resolved and can be fit to two populations with good accuracy and uniqueness. The equilibrium constants were derived from the EPR-determined populations.

Is it fair to compare the 10K EPR data with the room temperature data? Presumably the equilibrium constant is temperature dependent.

This topic has also been discussed in our prior papers using these NPs. The equilibrium constant is

quite temperature dependent,[1] as the reviewer surmised, but the equilibration between the states is quite slow, ca. 5-10 minutes. Control experiments showed that freezing and thawing of equilibrated and non-equilibrated samples did not change the **Red**/**Blue** ratio.[1,3] A sentence has been added (page 2, column 1) to address this very valid concern.

The change in the proton concentration for the blue was 50-100% larger than for the red; doesn't this imply that the stoichiometry for the blue is less than 2? Random error would give values > 100% if it was exactly 2. How does one rule out a distribution of proton stoichiometries for the blue? The physical location of the trap states may not be accessible surface sites, as was suggested, especially if the anatase polymorph is present that has internal channels that can host protons

We apologize for this confusion. The 50-100% $\Delta[H^+]$ referred to the change in protons relative to the *total* electron concentration (both red and blue) and not just compared to the one set of states. This section has been extensively revised to address concerns raised by other reviewers. We feel that the revision has clarified this issue.

Reviewer 3

Recommendation: Publish with minor revisions

Additional Questions:

Quality of experimental data, technical rigor: Top 5%
Significance to chemistry researchers in this and related fields: Top 1%
Broad interest to other researchers: Top 5%
Novelty: Top 5%
Is this research study suitable for media coverage or a First Reactions (a News & Views piece in the journal)?: Yes

Comments:

This study demonstrates that the different traps in TiO2 nanocrystals (previously deemed "red" and "blue" by the same group) have different proton stoichiometries. Specifically, it is shown that the two traps are in protic equilibrium, where adding a proton to the red trap yields a blue trap, which behaves analogously to a soluble molecular acid. This work highlights the importance of considering chemical stoichiometries in addition to electronic/structural properties when describing traps in semiconductor materials and provides a platform for understanding reactivity at metal oxide surfaces. It is thus of interest to the broad semiconductor and nanocrystal communities and is recommended for publication in ACS Central Science. One minor revision is suggested below.

We thank the reviewer for these comments.

It is very clearly demonstrated that the red trap has one less proton than the blue trap. The 2:1 ratio, however, was not quite as clear. It seems like this was claimed based on the data presented in Figure 3. It is stated that "the model of eqs 5 and 6 most closely matches behavior of the experimental data." However, the experimental data (blue circles) only matches this model (black dashed line) at one data point (~50% blue). If the trend of the experimental data is extended to lower % blue, it would cross the model for 1 H+ on blue and 0H+ on red (purple dashed line). I understand that a perfect agreement is not expected due to buffering from the nanocrystals, but can the authors elaborate on how it was determined that the better match was to the 2:1 model? Perhaps collecting a couple data points at lower %blue would make this more clear.

We thank the reviewer for pushing us to clarify this point. Unfortunately, we are unable to extend the data to higher pH (lower %**Blue**) because of the instability of the TiO_2 colloid. The text has been revised to indicate that the 2:1 conclusion is not as well established as the $1H^+$ difference

between the two types of trap states (which is called "very clearly demonstrated" by the reviewer). Still, all five datapoints in Figure 3 show that *more* protons are released than the 2:1 model would predict. On that basis, the 1:0 proton model suggested by the reviewer is very unlikely. The revised text (copied below) emphasizes that the 2:1 conclusion is based primarily on absolute amount of H^+ liberated, not the slope of the best fit line of the five datapoints.

The $\Delta[H^+]$ vs. **%Blue** slope indicates 2.2H^+ per **Blue** e^-. The best integer-coefficients model for that slope is 2H^+ per Blue e^- and zero H^+ per Red e^-, because (as the reviewer noted) that line would come close to zero H^+ at 0% **Blue** (100% **Red**). However, that model predicts a much lower amount of H^+ released than is observed, and the 2:0 ratio contradicts the well-established "Blue has one more H^+ than Red" stoichiometry" from the equilibrium measurements.

The revised text also cites prior papers for TiO_2 NPs and films, including our work with citrate-capped NPs from pH 3 to 7, that all show a roughly 1H^+ per e^- slope at the higher pHs (Finklea has an excellent summary in his book chapter). These prior results support the conclusion of at least 1H^+ per e^- for reduction of TiO_2 NPs studied here and argue against the 0H^+ per e^- extrapolation to 100% **Red** suggested by the reviewer. We note that in general for the electrochemistry of metal oxides, super-Nernstian behavior (>59 mV/pH) is quite common while we know of no examples where the band shifts with pH are <59 mV/pH.

> With the assumption of integer stoichiometries for each trap state, the results are most consistent with stoichiometries of 2H^+ per **Blue** e^- and 1H^+ per **Red** e^- (eqs 5 and 6). The prediction of this model is shown as the dashed black line in Figure 3….
>
> The best fit line for the blue points in Figure 3 has a slope of 2.2 more protons per electron in **Blue** over **Red**. This might suggest a 2:0 model where the Blue states have two protons per electron while the Red states are not proton-coupled. However, this model (purple dashed line) does not fit the data closely, and it does not obey the conclusion above that **Blue** = **Red** + one proton. Thus, the stoichiometries in eqs 5 and 6 are the best model for the data.
>
> Still, the blue points in Figure 3 deviate from the 2H^+ per **Blue** e^- and 1H^+ per **Red** e^- model. The discrepancy is likely in part due to changes in the buffering of the TiO_2 upon oxidation, which is not included in the model.

REFERENCES

(1) Peper, J. L.; Vinyard, D. J.; Brudvig, G. W.; Mayer, J. M., Slow Equilibration between Spectroscopically Distinct Trap States in Reduced TiO_2 Nanoparticles. Journal of the American Chemical Society 2017, 139 (8), 2868-2871. https://doi.org/10.1021/jacs.6b12112

(2) Peper, J. L.; Gentry, N. E.; Boudy, B.; Mayer, J. M., Aqueous TiO_2 Nanoparticles React by Proton-Coupled Electron Transfer. Inorganic Chemistry 2022, 61 (2), 767-777. https://doi.org/10.1021/acs.inorgchem.1c03125

(3) Peper, J. L.; Gentry, N. E.; Brezny, A. C.; Field, M. J.; Green, M. T.; Mayer, J. M., Different Kinetic Reactivities of Electrons in Distinct TiO_2 Nanoparticle Trap States. The Journal of Physical Chemistry C 2021, 125 (1), 680-690. https://doi.org/10.1021/acs.jpcc.0c10633

(4) Peper, J. L. Studies of Titanium Dioxide Nanoparticles: Thermodynamics and Reactivity. Yale University, New Haven, Connecticut, 2019.

(5) Justin L. Lee, J. L. P., Noreen E. Gentry, Staci Hetzel, James M. Mayer, Oxygen Atom Transfer To and From Colloidal Metal Oxide Nanoparticles. Manuscript in preparation

(6) Nedzbala, H. S.; Westbroek, D.; Margavio, H. R. M.; Yang, H.; Noh, H.; Magpantay, S. V.; Donley, C. L.; Kumbhar, A. S.; Parsons, G. N.; Mayer, J. M., Photoelectrochemical Proton-Coupled Electron Transfer of TiO_2 Thin Films on Silicon. Journal of the American Chemical Society 2024, 146 (15), 10559-10572. https://doi.org/10.1021/jacs.4c00014

(7) Chiesa, M.; Paganini, M. C.; Livraghi, S.; Giamello, E., Charge trapping in TiO_2 polymorphs as seen by Electron Paramagnetic Resonance spectroscopy. Physical Chemistry Chemical Physics 2013, 15 (24), 9435-9447. http://dx.doi.org/10.1039/C3CP50658D

(8) Howe, R. F.; Gratzel, M., EPR observation of trapped electrons in colloidal titanium dioxide. The Journal of Physical Chemistry 1985, 89 (21), 4495-4499. https://doi.org/10.1021/j100267a018

(9) Finklea, H. O., Titanium Dioxide (TiO_2) and Strontium Titanate ($SrTiO_3$). In Semiconductor Electrodes, Elsevier: 1988; Chapter 2.

(10) Moss-Burnstein Effect, https://en.wikipedia.org/wiki/Moss%E2%80%93Burstein_effect, (accessed September 9 2024).

(11) Burke, L. D.; Lyons, M. E.; O'Sullivan, E. J. M.; Whelan, D. P., Influence of hydrolysis on the redox behaviour of hydrous oxide films. Journal of Electroanalytical Chemistry and Interfacial Electrochemistry 1981, 122, 403-407. https://www.sciencedirect.com/science/article/pii/S0022072881801763

(12) Minella, M.; Maurino, V.; Minero, C.; Pelizzetti, E., Thin Film Nanocrystalline TiO_2 Electrodes: Dependence of Flat Band Potential on pH and Anion Adsorption. Journal of Nanoscience and Nanotechnology 2015, 15 (5), 3348-3358. https://www.ingentaconnect.com/content/asp/jnn/2015/00000015/00000005/art000050 https://doi.org/10.1166/jnn.2015.10206

(13) Stoerzinger, K. A.; Rao, R. R.; Wang, X. R.; Hong, W. T.; Rouleau, C. M.; Shao-Horn, Y., The Role of Ru Redox in pH-Dependent Oxygen Evolution on Rutile Ruthenium Dioxide Surfaces. Chem 2017, 2 (5), 668-675. https://www.sciencedirect.com/science/article/pii/S2451929417301365

(14) Lyon, L. A.; Hupp, J. T., Energetics of the Nanocrystalline Titanium Dioxide/Aqueous Solution Interface: Approximate Conduction Band Edge Variations between $H_0 = -10$ and $H_- = +26$. The Journal of Physical Chemistry B 1999, 103 (22), 4623-4628. https://doi.org/10.1021/jp9908404

(15) Mayer, J. M., Bonds over Electrons: Proton Coupled Electron Transfer at Solid–Solution Interfaces. Journal of the American Chemical Society 2023, 145 (13), 7050-7064. https://doi.org/10.1021/jacs.2c10212

(16) Noh, H.; Mayer, J. M., Medium-independent hydrogen atom binding isotherms of nickel oxide electrodes. Chem 2022, 8 (12), 3324-3345. https://www.sciencedirect.com/science/article/pii/S2451929422004314

oc-2024-01074y.R2

Name: Peer Review Information for "Trap States in Reduced Colloidal Titanium Dioxide Nanoparticles Have Different Proton Stoichiometries"

Second Round of Reviewer Comments

Reviewer: 2

Comments to the Author

The authors provided a very detailed response which is greatly appreciated.

I remain however lukewarm on the importance of publishing such work in a high impact journal like ACS Central Science as the study is limited to the small TiO_2 nanoparticles formed from $TiCl_4$ that is known to have amorphous TiO_2 and (reportedly) some anatase. Hupp reported long ago that for anatase TiO_2 it is a one electron, one proton reduction over 13 decades of proton activity. It may well be that the more poorly defined "TiO_2" has a different stoichiometry over the very limited pH range studied here, yet this stoichiometry was already reported in JACS (2017) and may not be of broader interest.

The authors response indicated that "We respectfully disagree that “the vast majority of SC interfaces, and arguably the most important technologically, are not with aqueous solutions.” I believe it is just a different point of view. Real world applications of semiconductors exist in integrated circuits, transistors, solar cells, sensors, and others to the tune of ~ 530 billion dollars/year. These clearly do not have an aqueous interface and the physical models given in standard texts like Sze are appropriate. Fujashima and Honda's studies with rutile TiO_2 clearly involve an aqueous interface and there has been continued funding for solar water splitting that drives academic interests even though no applications have been forthcoming. I think the authors recognize this and the tone was indeed softened.

Author's Response to Peer Review Comments:

• A revised manuscript file including a synopsis is here uploaded.

• A file explaining the author addition, signed by all of the authors, is here uploaded as an “Other Files for Editors Only” file.

Thank you for your attention to this manuscript.
